# Supplementary material for: Anion redox as a means to derive layered manganese oxychalcogenides with exotic intergrowth structures
Source: Nat Commun. 2023 May 22;14:2917. doi: 10.1038/s41467-023-38489-3 (PMC10202913; doi:10.1038/s41467-023-38489-3)
Supplement: Supplementary file 1 — Supplementary Information [file 41467_2023_38489_MOESM1_ESM.pdf]

Supplementary Information

Anion Redox as a Means to Derive Layered manganese Oxychalcogenides with Exotic Intergrowth Structures

Sasaki et al.

Supplementary Figures

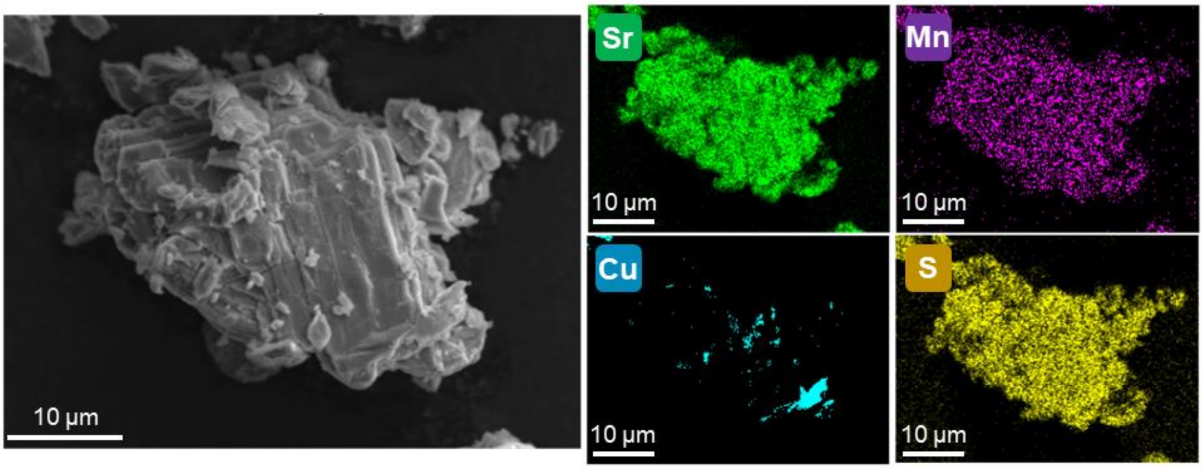

Supplementary Figure 1. SEM analyses of $Sr_2MnO_2Li_{1.9}S_2$ + 1.5 Cu. (Left) The secondary electron (SE) image of the product which was obtained by the reaction of $Sr_2MnO_2Cu_{1.5}S_2$ with *n*-BuLi at 50 °C (Step 1, see Figure 1a in the main article). (Right) element mapping displaying the signals from Sr $L\alpha_1$ (green), Mn $K\alpha_1$ (purple), Cu $L\alpha_{1,2}$ (cyan) and S $K\alpha_1$ (yellow), respectively. Acceleration voltage was limited to 10 kV for better resolution of element mapping. Cu is clearly extruded onto the surface.

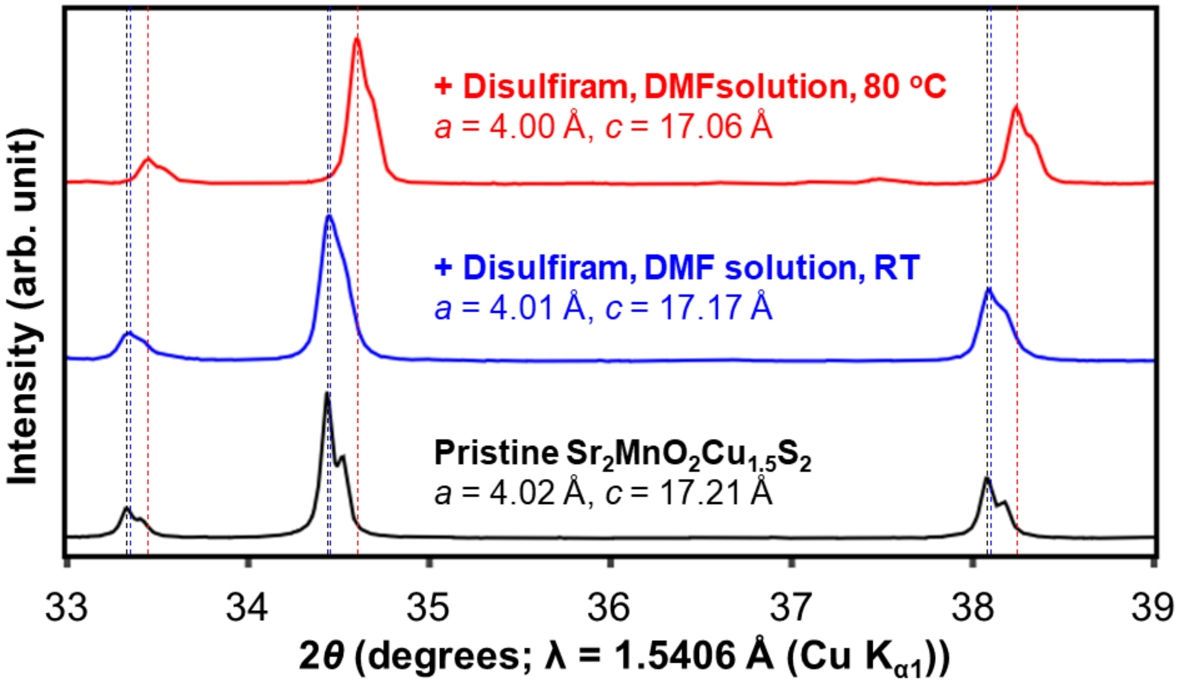

Supplementary Figure 2. X-ray diffraction (XRD) patterns after the reaction of $Sr_2MnO_2Cu_{1.5}S_2$ with disulfiram. Zoom-in views of the laboratory powder XRD patterns of pristine $Sr_2MnO_2Cu_{1.5}S_2$ (black) and the products after its treatments with excess (6.0 equiv.) of disulfiram in DMF solution at ambient temperature (blue) and at 80 °C (red). Rietveld refinements using the respective patterns indicated that the treatment with disulfiram led to small cell contraction of the $Sr_2MnO_2Cu_{1.5}S_2$ structure model, but to the lesser extents than $Sr_2MnO_2Cu_{1.5-x}S_2$ phase ($x < 0.17$) reported by Blandy et al.[1]

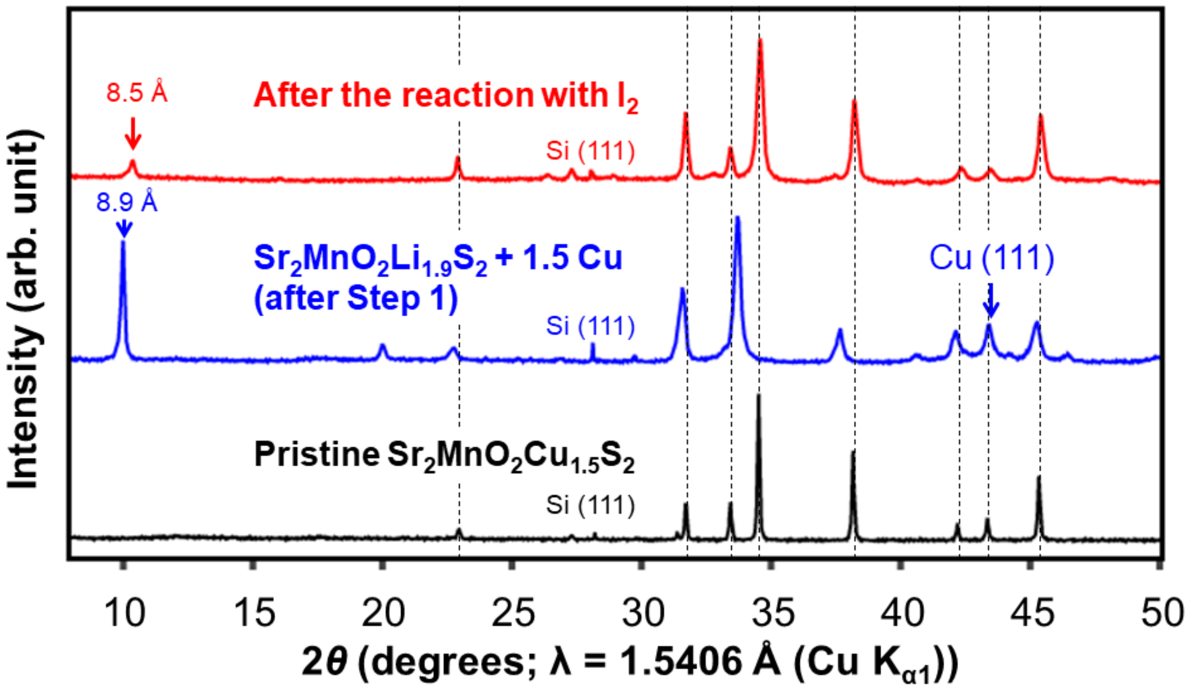

Supplementary Figure 3. X-ray diffraction (XRD) patterns after attempted Cu dissolution using I_2. The pristine parent phase $Sr_2MnO_2Cu_{1.5}S_2$ (black pattern) was first converted to $Sr_2MnO_2Li_{1.9}S_2$ + 1.5 Cu using *n*-BuLi (Step 1 reaction in Figure 1a; blue pattern). Then, the reaction mixture was treated with 2.0 equiv. of I_2 (15 mM solution of anhydrous acetonitrile) at 0 °C, overnight, resulting in the recovery of the parent phase (red pattern) instead of Cu dissolution. Rietveld refinement of the product structure indicated its unit cell was slightly smaller (a = b = 4.00 Å, c = 17.09 Å) than the pristine parent phase (a = b = 4.02 Å, c = 17.21 Å). The cell contraction, together with its more pronounced 002 reflection at d = 8.5 Å, are ascribed to off-stoichiometry of Cu in $Sr_2MnO_2Cu_{1.5-x}S_2$ phase (x < 0.17) as reported by Blandy et al.[1]

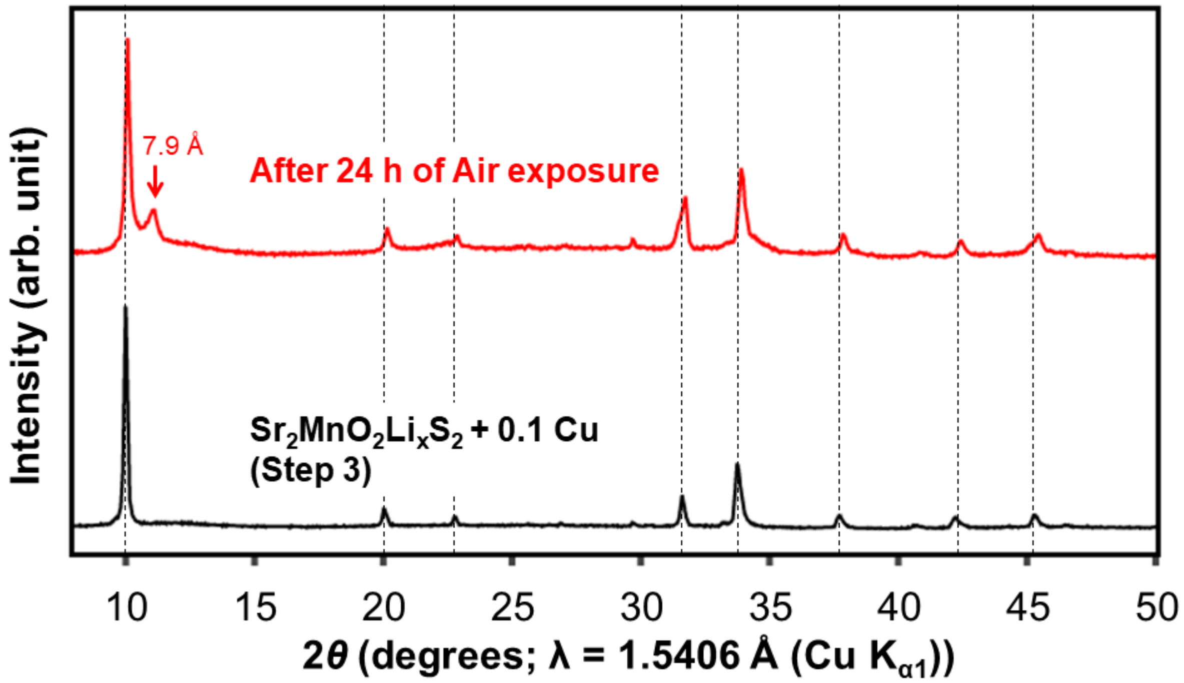

Supplementary Figure 4. X-ray diffraction (XRD) patterns of the lithiated Step 3 products before and after air exposure. Air exposure of the Step 3 product $Sr_2MnO_2Li_xS_2$ + *y* Cu (*x* ~ 1.9, *y* ~ 0.1) led to emergence of the new peak at around 7.9 Å, indicating the formation of the collapsed phase as a minor phase.

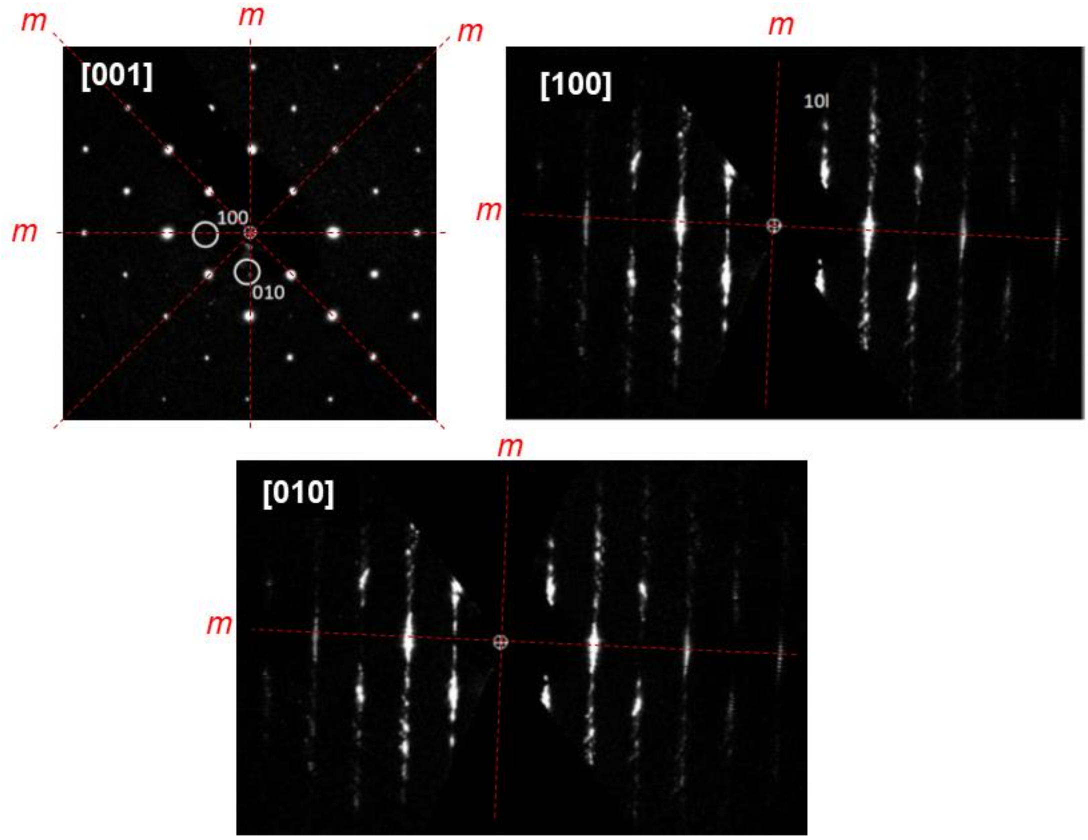

Supplementary Figure 5. Three-dimensional electron diffraction (3D ED) analysis of the collapsed oxysulfide phase. The reconstructed [001] (top, left), [100] (top, right) and [010] (bottom) section from electron diffraction tomography (EDT). Red broken lines represent hypothetical mirror planes in the case where *I*4/*mmm* space group is assumed. The [001] zone complied with the reflection condition $h + k = 2n$ of *I*- or *C*-centered lattices. Also [100] and [010] displayed a weak tendency toward *I*-centering but their diffraction spots were severely blurred by diffuse streaks, indicating disorder along the *c*-direction. Sample 1 (bulk composition estimated by ICP-MS: $Sr_{2.00(2)}Mn_{1.07(2)}O_2Cu_{0.35(0)}Li_{0.13(0)}S_2$) was used for the analysis.

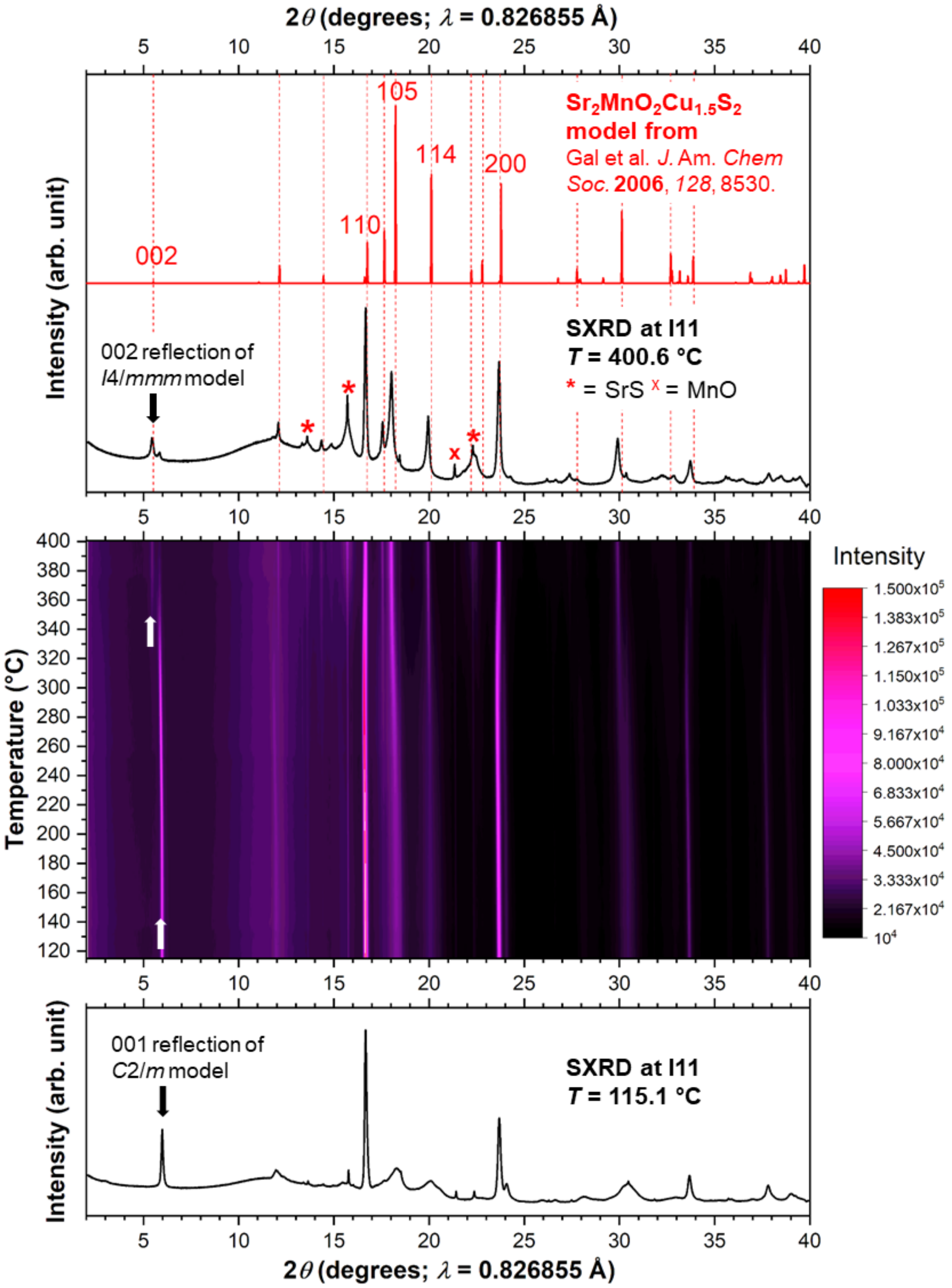

Supplementary Figure 6. Variable-temperature (VT) synchrotron X-ray diffraction (SXRD) of the collapsed oxysulfide phase. The colour plot shows the transition of synchrotron powder XRD patterns as a function of temperature from *T* = 115.1 °C (bottom) to 400.6 °C (top). Above 300 °C, the peaks from the collapsed phase are accompanied by those expected for the $Sr_2MnO_2Cu_{1.5}S_2$ phase[2] together with binary decomposition phases. See Supplementary Figure 7.

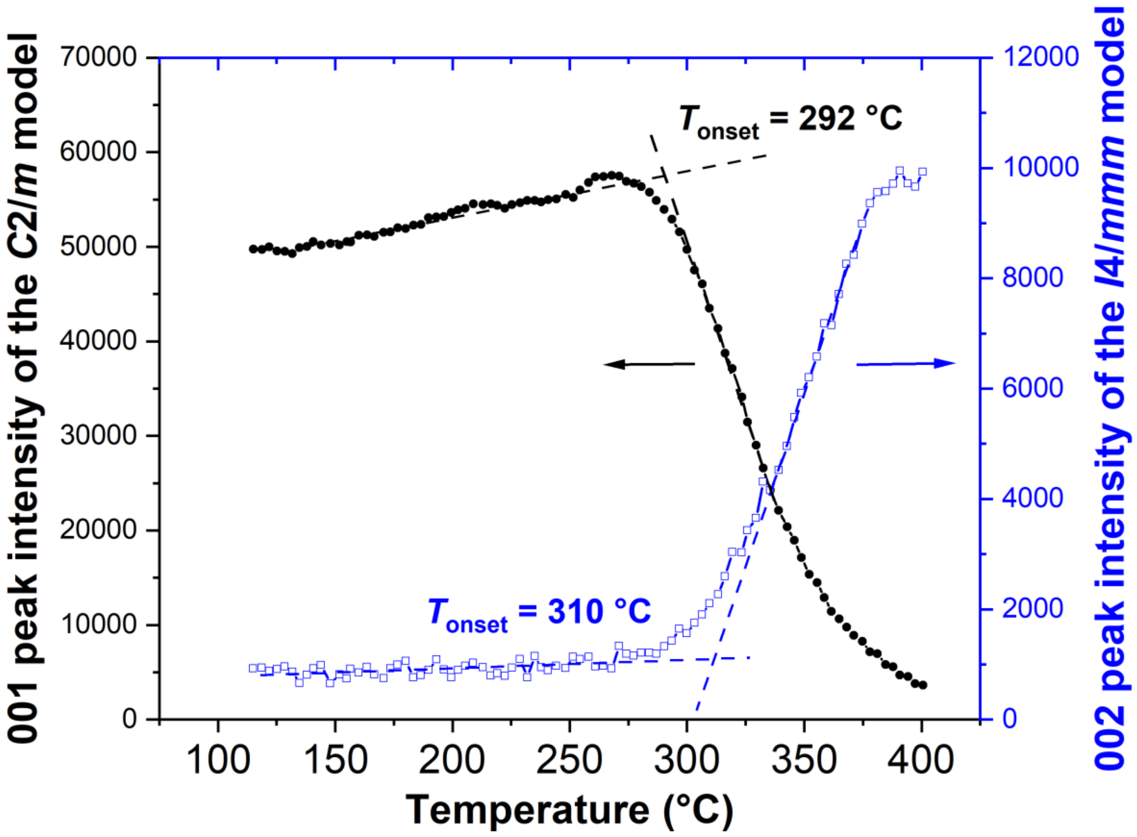

Supplementary Figure 7. Intensity profile of 001 reflection of the collapsed *C*2/*m* model (black) and 002 reflection of the parent *I*4/*mmm* model (blue). The *C*2/*m* model is depicted in Figure 2b and the *I*4/*mmm* model corresponds to the structure of $Sr_2MnO_2Cu_{1.5}S_2$ phase reported by Gál et al.[2] Each of these peaks was highlighted by black arrows in the top and bottom panel of Supplementary Figure 6. Background signals were approximated by linear interpolation at respective peak positions and subtracted from the observed intensity.

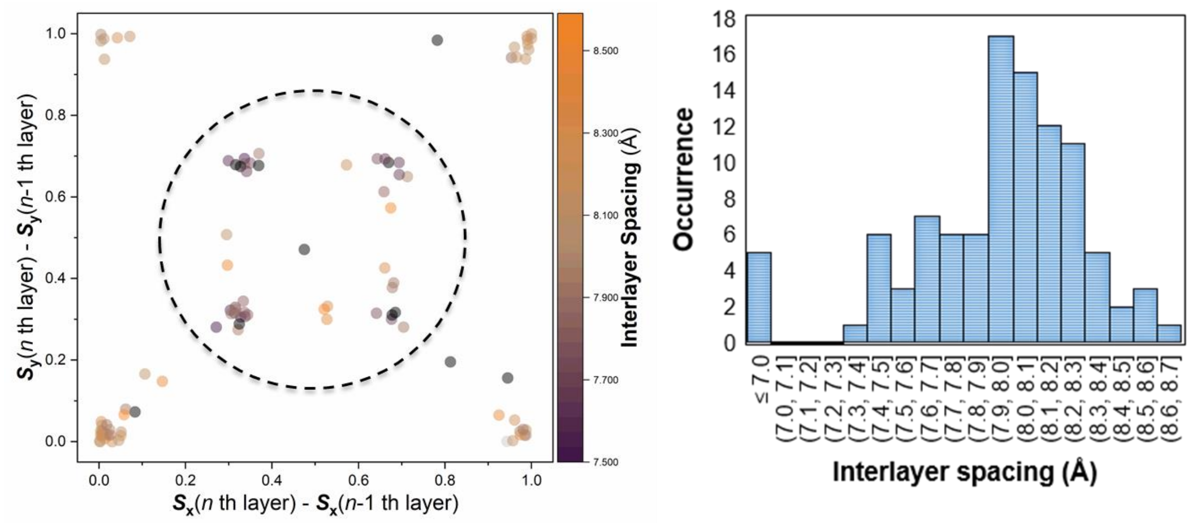

Supplementary Figure 8. Plot representing *xyz* shifts of $Sr_2MnO_2S_2$ stacks after Rietveld refinement against Synchrotron XRD data (See Figure 2a). (Left) Scatter plots displaying sliding of each layer in *xyz* directions (S_x, S_y, S_z) that was refined as fractional values of unit cell parameters. Sliding along the stacking axis S_z was converted into interlayer spacing between adjacent $Sr_2MnO_2S_2$ layers and shown as a function of colour. The slabs that slide along the diagonal direction (encircled by the broken line) exhibited a tendency toward smaller interlayer spacing. (Right) Histogram showing the occurrence of each interlayer spacing.

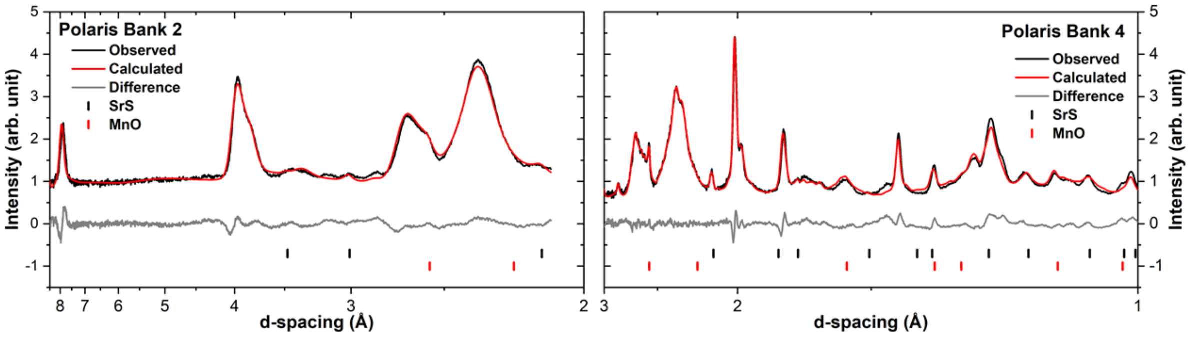

Supplementary Figure 9. Rietveld fits to time-of-flight (TOF) powder neutron diffraction (PND) data of the final product of multistep Cu deintercalation shown in Figure 1a. PND data is shown from Bank 2 (2θ = 25.99°) and Bank 4 (2θ = 92.59°) of the POLARIS diffractometer at the ISIS pulsed neutron source.[3] Refinement was carried out for the same structure model with the one described in Figure 2b (See Section 3.2 of the SI for the details). For the plots TOF data was converted into *d*-spacing scale using TOF = $t_0 + t_1 d + t_2 d^2$, where zero-time offset t_0, instrumental parameter t_1 and absorption-related correction t_2 were obtained from the most recent calibration using Si powder (NIST SRM 640b (a = 5.43094Å)) and $Y_3Al_5O_{12}$ (YAG) powder. Data (black), fit (red), difference (grey) were plotted and tick marks represent Bragg peak positions of the impurity phases. R_{wp} = 2.61% was given as the result of fitting both diffraction patterns at the same time.

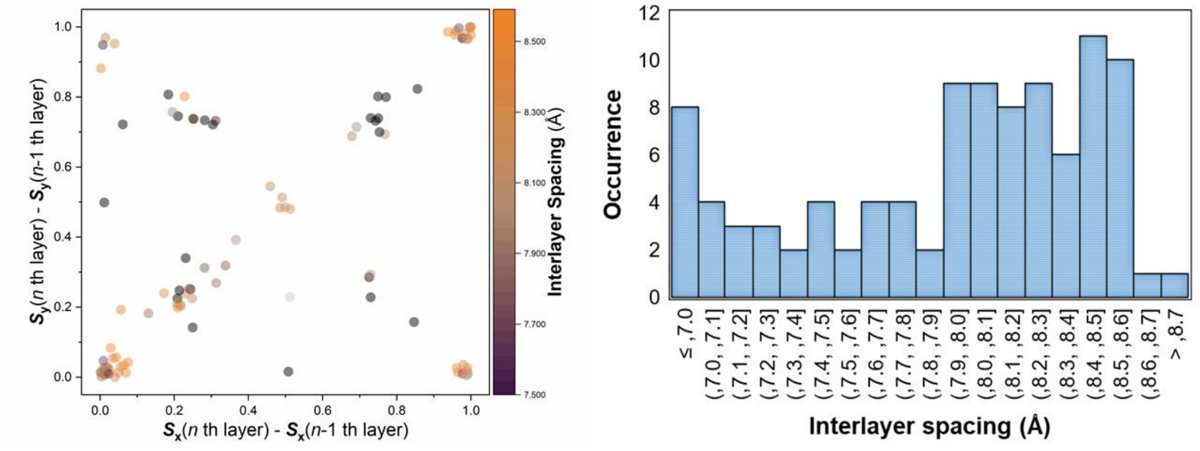

Supplementary Figure 10. Plot representing *xyz* shifts of $Sr_2MnO_2S_2$ stacks after Rietveld refinement against PND data (See Supplementary Figure 9). (Left) Scatter plots displaying sliding of each layer in *xyz* directions (S_x, S_y, S_z) that was refined as fractional values of unit cell parameters. Sliding along the stacking axis S_z was converted into interlayer spacing between adjacent $Sr_2MnO_2S_2$ layers and shown as a function of colour. In the same way with the case of XRD (Supplementary Figure 8), the slabs that slide along the diagonal direction exhibited tendency toward smaller interlayer spacing. (Right) Histogram showing the occurrence of each interlayer spacing.

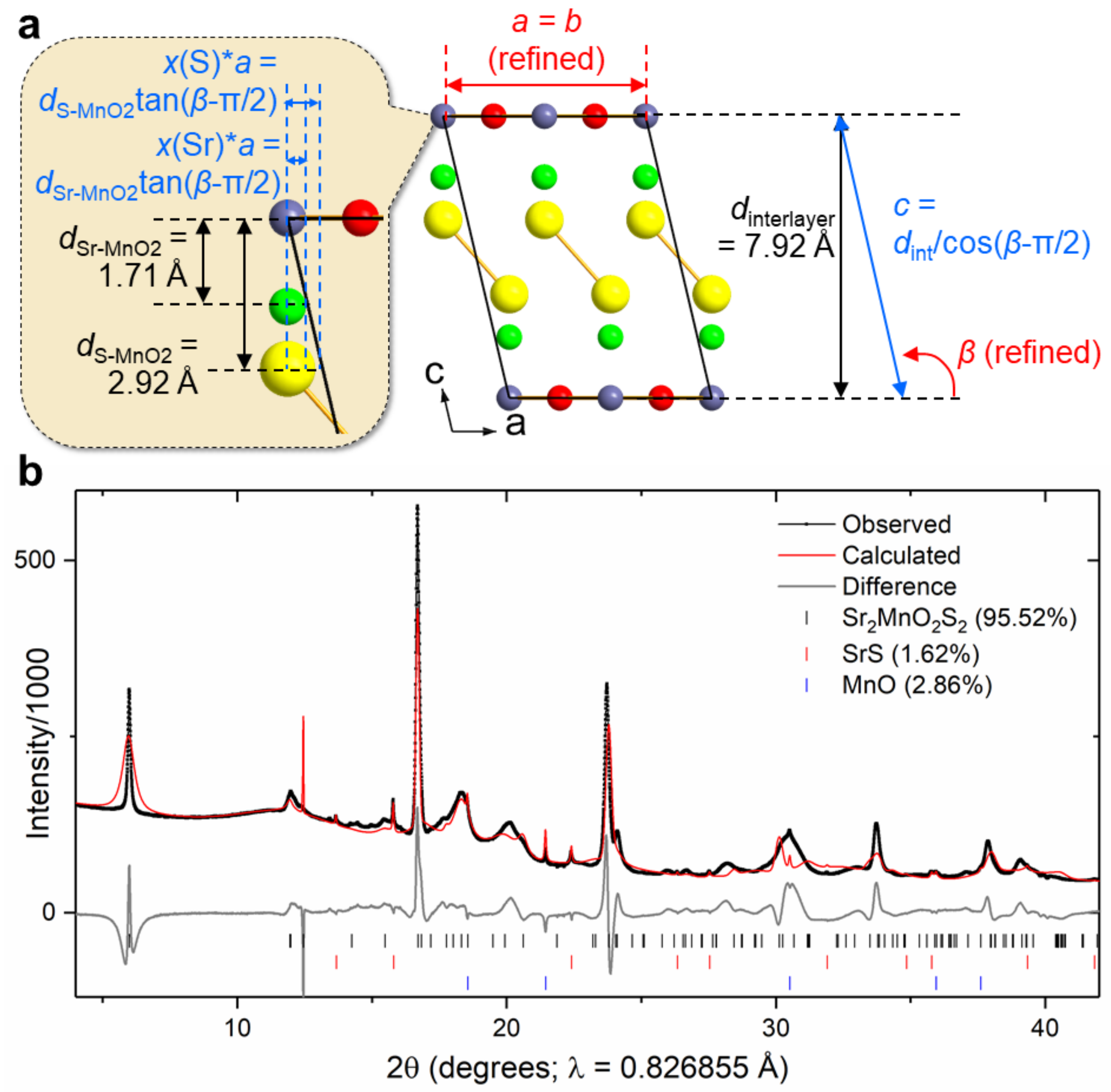

Supplementary Figure 11. Tentative Rietveld refinement of the collapsed phase using a *C*2/*m* model fixing the structural parameters of the $Sr_2MnO_2S_2$ slab, which can be arrived at by removing the copper from the parent structure and shifting on $Sr_2MnO_2S_2$ slab by 0.97 Å in the 110 direction relative to the layer below. **a** The structure model used for the refinement (Space group: *C*2/*m*). Fixed parameters, refined parameters and constrained parameters were designated respectively in black, red and blue. The structural parameters of the $Sr_2MnO_2S_2$ slab was fixed to those taken from the $Sr_2MnO_2Cu_{1.5}S_2$ model reported by Gál et al;[2] Perpendicular distance from MnO_2 plane to Sr and S atoms were fixed to $d_{Sr\text{-}MnO2}$ = 1.71 Å and $d_{S\text{-}MnO2}$ = 2.92 Å, respectively. The interlayer spacing was fixed to the position of the hypothetical 001 reflection (d = 7.92 Å), and accordingly the c parameter was constrained as a function of the refined β angle. **b** The Rietveld fit to Synchrotron XRD data (R_{wp} = 11.5%, GOF = 36.0). The refined cell parameter $a = b$ = 5.69 Å and β = 103.38° qualitatively explained the experimental PXRD pattern (See Figure 2b in the main article), but the poor fitting implied that the structure modelling must be further improved, e.g. taking stacking faults into account (See main article). During the refinement, anisotropic microstrain was taken into account employing Stephens's tensor method.[4]

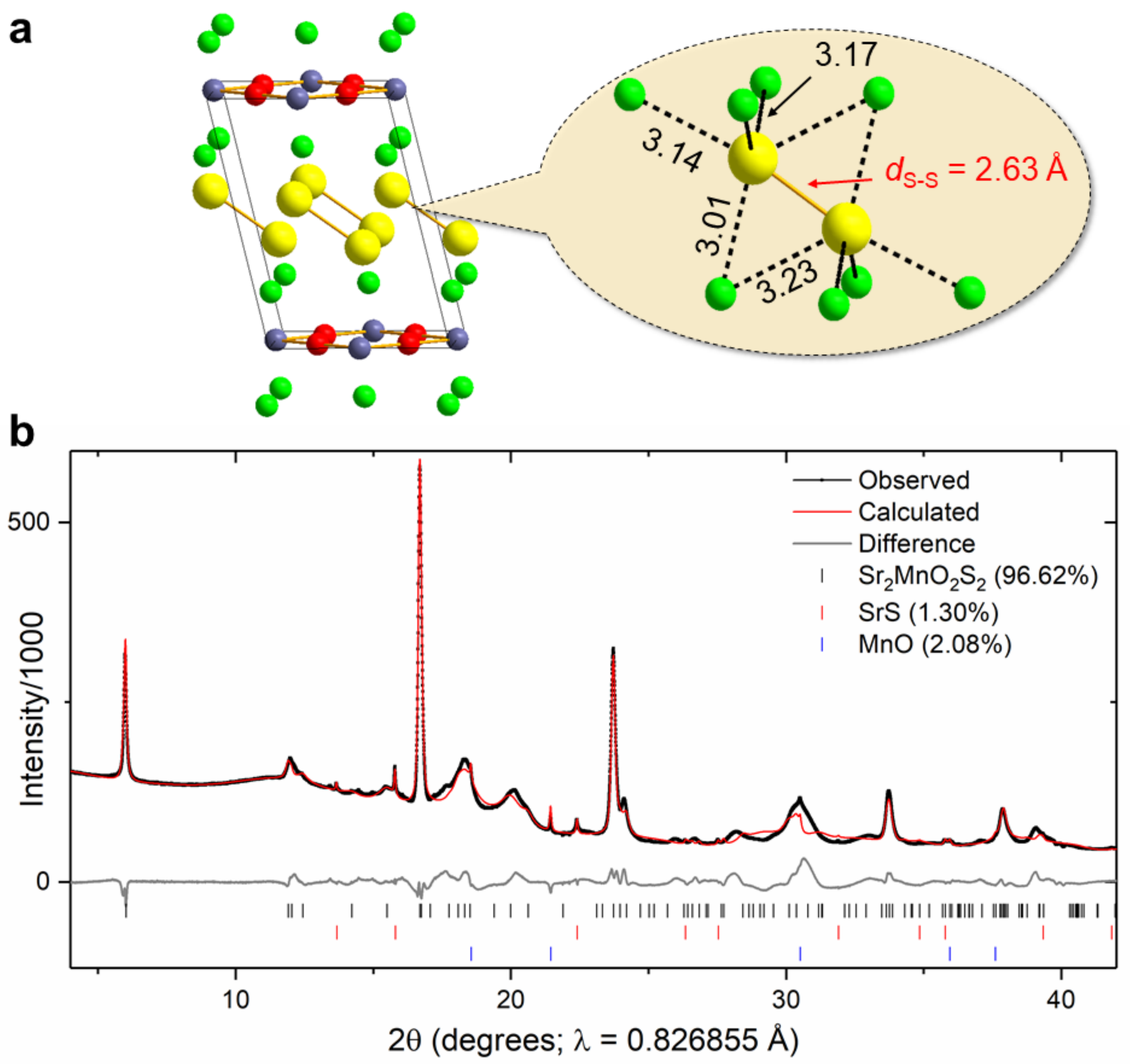

Supplementary Figure 12. Tentative Rietveld refinement of the collapsed phase using the *C*2/*m* model allowing its atomic positions to be refined without constraint. a The refined *C*2/*m* model: *a* = 5.72 Å, *b* = 5.70 Å, *c* = 8.11 Å, *β* = 103.2°, Sr(*x*,*y*,*z*) = (0.089, 0, 0.227), Mn(*x*,*y*,*z*) = (0, 0.5, 0), O(*x*,*y*,*z*) = (0.25, 0.25, 0), S(*x*,*y*,*z*) = (0.655, 0, 0.403). (Inset) Bond lengths (Å) around the S_2 unit. Note that the refined S-S bond length of 2.63 Å is too long to assume a S-S single bond and therefore it is likely that the value does not reflect the real structure. **b** The Rietveld fit to Synchrotron XRD data (R_{wp} = 5.6%, GOF = 17.6). The quality of fitting was significantly improved from the one shown in Supplementary Figure 11, but the calculated pattern still exhibited serious deviation from the experimental pattern. Stacking faults must be taken into account for further improvement of the fitting (See the main article). During the refinement, anisotropic microstrain was taken into account employing Stephen's tensor method.[4]

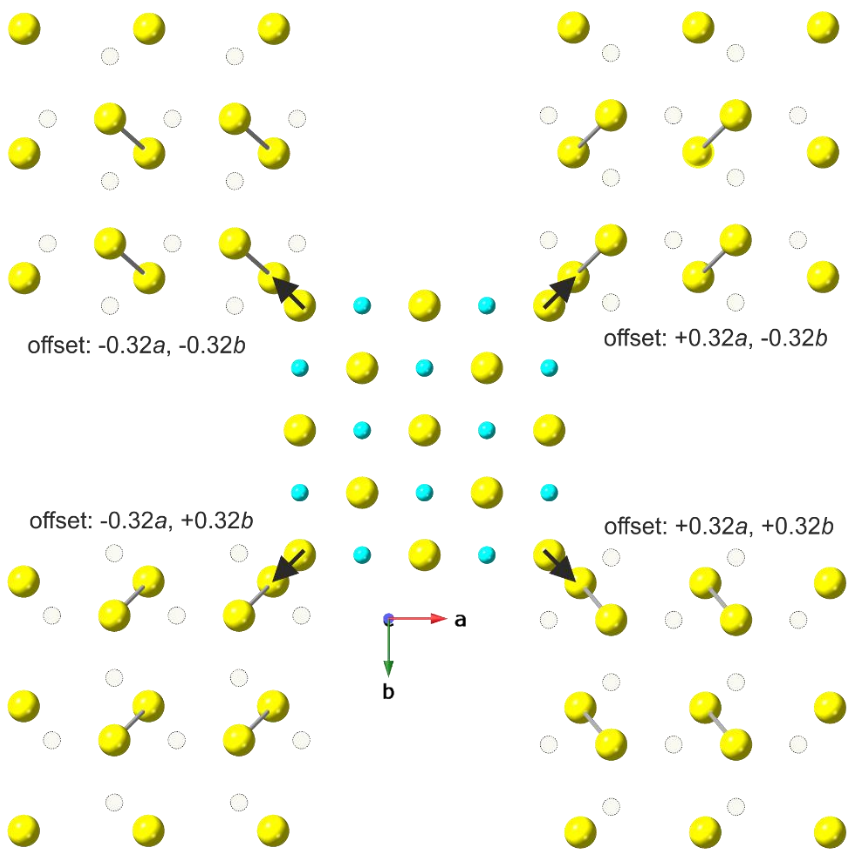

Supplementary Figure 13. Four possible displacements on the removal of copper from the layers leading to the formation of S-S bonds. A *C*2/*m* symmetry structure could be arrived at if only a single choice of offset is chosen throughout the crystal. A change from one offset to another can be thought of as akin to crystal twinning. The STEM in Figure 5 shows there are multiple switches in S-S bond direction, giving a highly faulted structure.

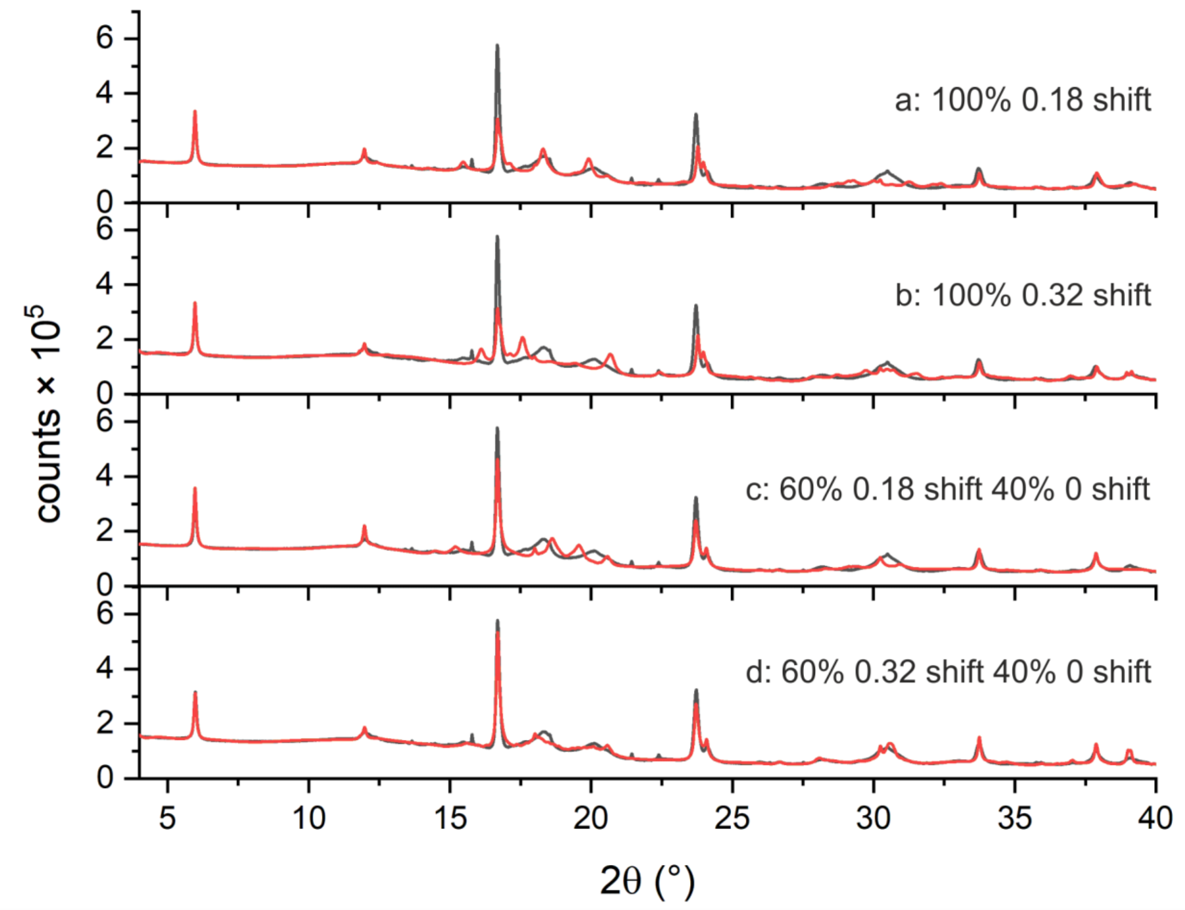

Supplementary Figure 14. Rietveld fits to the PXRD data of the collapsed phase using a stacking fault model of 1000 layers as the base unit cell. In each case the 1000-layer-cell's composition and stacking distribution is determined by the probabilities s, P_0, P_1,P_2,P_3, and P_4. **a**: s = 0.17, P_0 = 0, P_1 = 0.76, P_2=P_3=P_4 = 0.08. **b**: s = 0.32, P_0 = 0, P_1 = 0.76, P_2=P_3=P_4 = 0.08. **c**: s = 0.17, P_0 = 0.4, P_1 = 0.456, P_2=P_3=P_4 = 0.048. **d**: s = 0.32, P_0 = 0.4, P_1 = 0.456, P_2=P_3=P_4 = 0.048. See Supplementary Note 1.

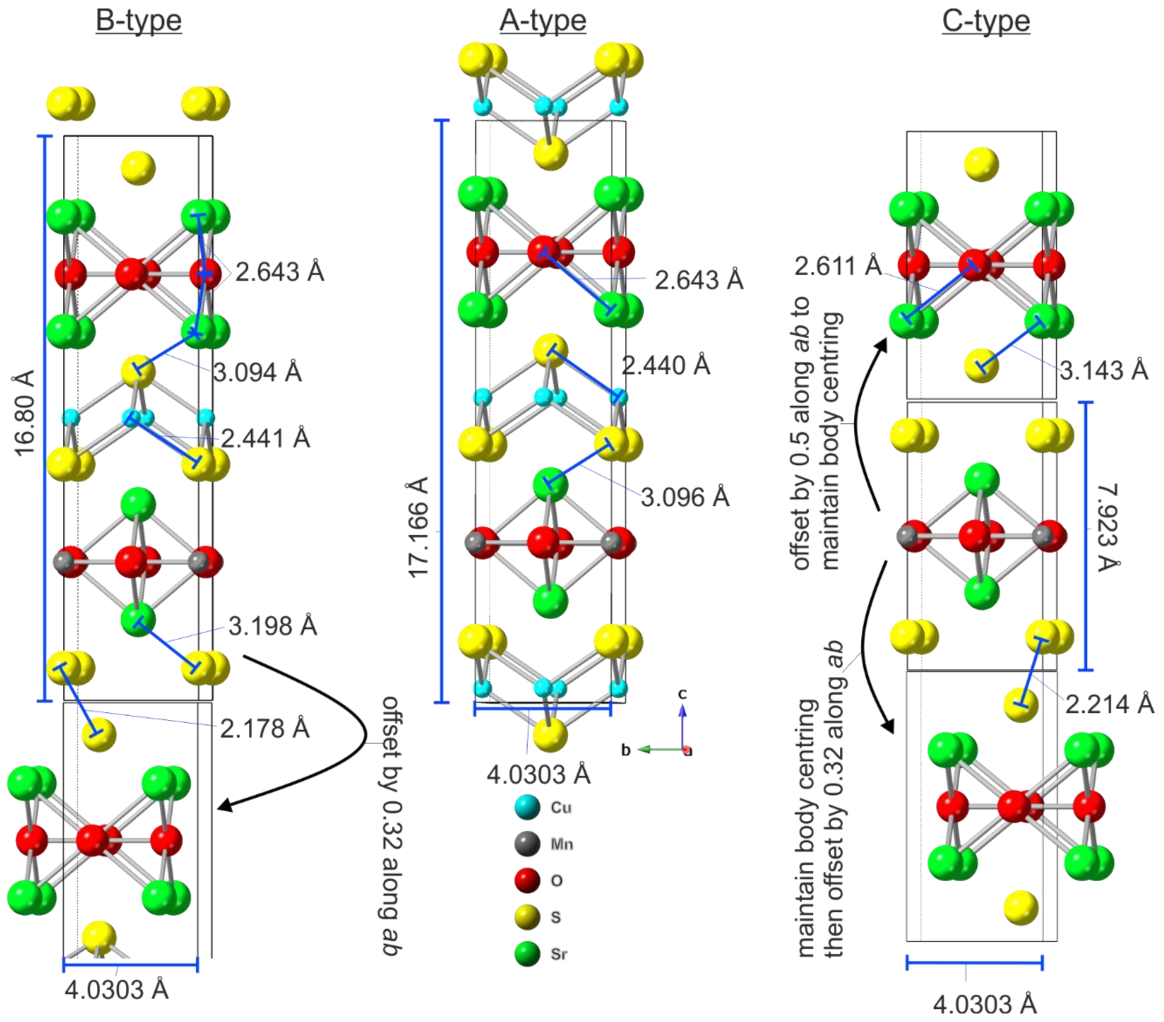

Supplementary Figure 15. Defined structures and bond distances of the A-type (parent), B-type (intergrowth), and C-type (collapsed) phases, which are used as a basis for the stacking fault modelling in of the collapsed sulfide phase.

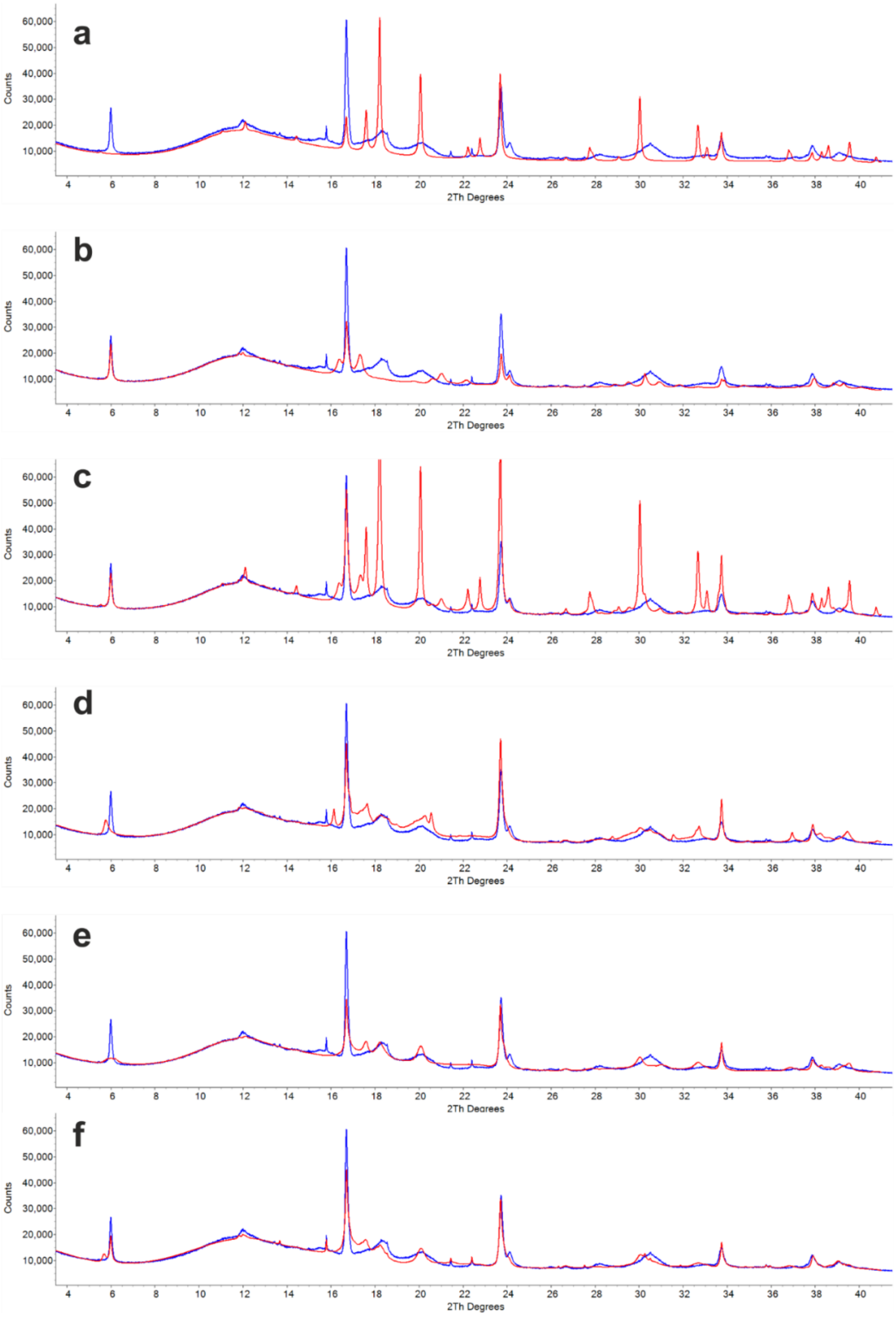

Supplementary Figure 16. Rietveld fits to powder XRD data from the collapsed sulfide phase using 1500-layer supercells defined with parameters *s*, $P_{AA\text{-}CC}$, P_m, and P_1. a: A-type only, no faults. **b**: C-type only, with an intermediate level of twinning faults (P_1=0.8). **c**: A-type and C-type intergrown with a low P_m value corresponding to a 99.5% chance of both A-A and C-C. **d**: A-type and C-type intergrown with a high P_m value corresponding to a 40% chance of A-A and 60% chance of C-C. **e**: A-type intergrown with C-type that has a shortened c lattice parameter (7.7 Å), with a P_m value corresponding to a 60% chance of A-A and 80% chance of C-C. **f**: two supercell phases: 1st phase C-type only with high twinning faults (P_1=0.4), 2nd phase: A-,B-, and C-type with low twinning faults (P_1 = 0.95), highly intergrown: AA=0.55, AB=0.15, AC=0.30, BA=0.47, BB=0.05 ,BC=0.48, CA=0.31, CB=0.16, CC=0.53. See Supplementary Note 1.

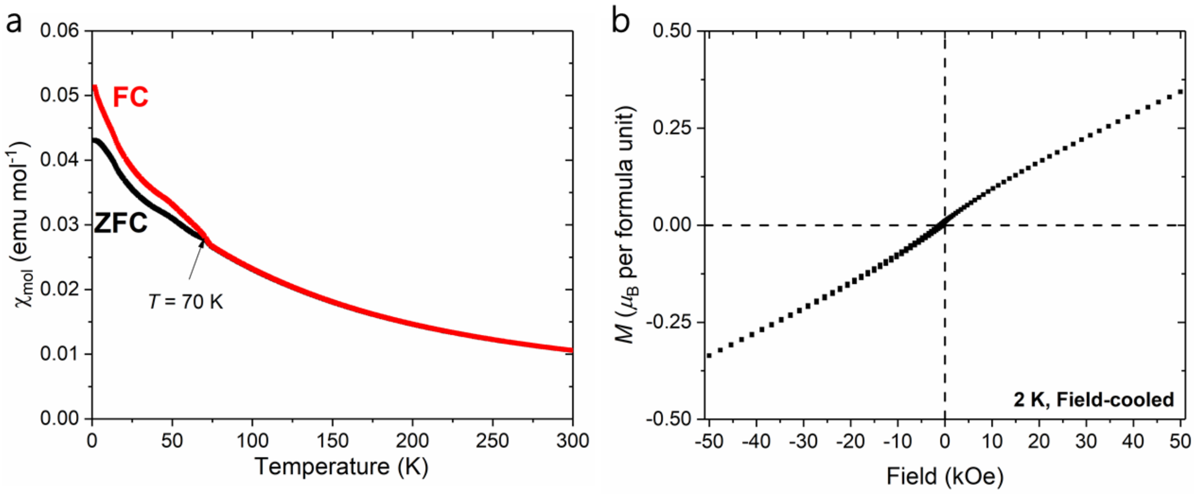

Supplementary Figure 17. χ_{mol} versus *T* plot and magnetisation isotherm of the collapsed oxysulfide phase. a Zero-field-cooled (ZFC, black) and Field-cooled (FC, red) magnetic susceptibilities of the collapsed phase measured in an applied field of 0.01 T. Magnetisation (emu) was converted into χ_{mol} (emu mol^{-1} Oe^{-1}) supposing the tentative formula $Sr_2MnO_2S_2$ (Molar mass = 326.31 g mol^{-1}). In the same way with partially deintercalated $Sr_2MnO_2Cu_{1.33}S_2$ phase,[1] the collapsed phase exhibited slight divergence between ZFC and FC susceptibilities at higher temperature (T = 70 K) than the parent phase (T_N around 30 K)[3]. **b** Magnetisation isotherm measured at 2 K after cooling at H = 50 kOe. Molar magnetisation M was calculated supposing the tentative formula $Sr_2MnO_2S_2$ (Molar mass = 326.31).

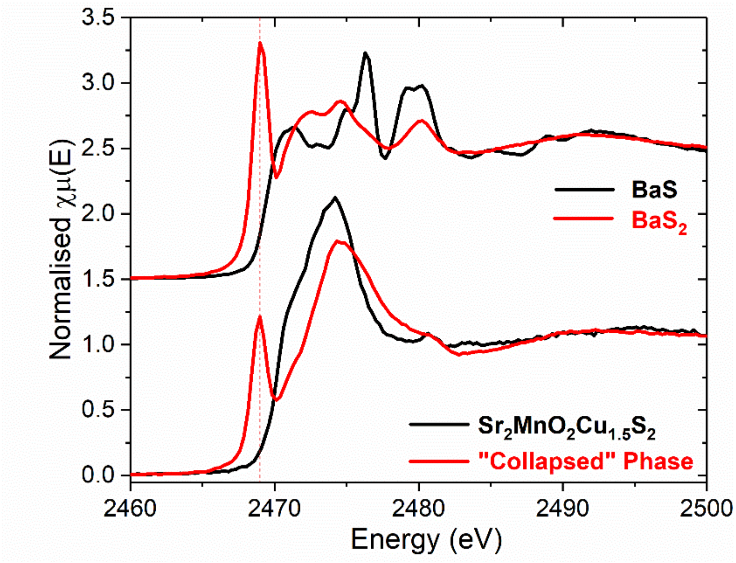

Supplementary Figure 18. Sulphur K-edge X-ray absorption near-edge structure (XANES) spectra. (Top) the XANES spectra of BaS and BaS_2; (bottom) the XANES spectra of the parent $Sr_2MnO_2Cu_{1.5}S_2$ phase and the collapsed oxysulfide phase. BaS was purchased from a commercial source (Aldrich, 99.9%) and BaS_2 was synthesised from BaS following the method reported elsewhere.[5] The dotted line at 2469 eV points to the position of the pre-edge peaks of oxidised sulfide anions.

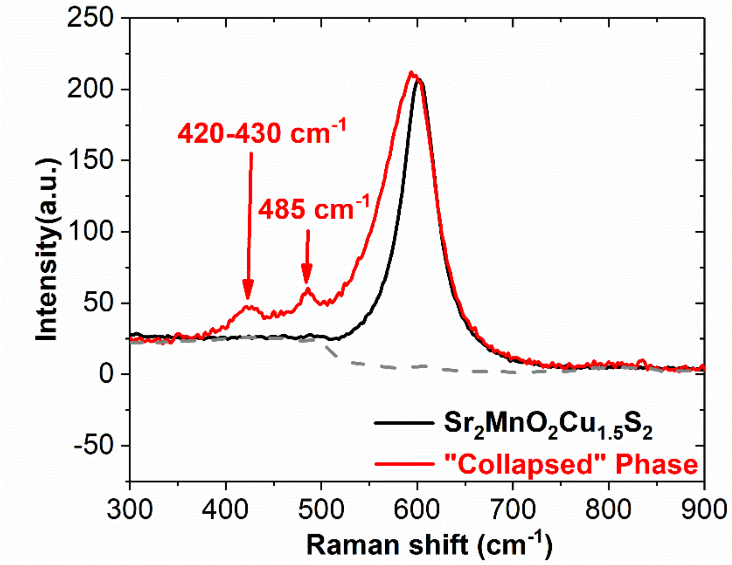

Supplementary Figure 19. Raman spectra of $Sr_2MnO_2Cu_{1.5}S_2$ and its collapsed phase measured at excitation wavelength λ_{ex} = 785 nm under inert atmosphere. Both powder sample were packed and loaded into quartz capillaries under argon. Raman spectrum of the empty quartz capillary (grey dotted line) was also measured as a blank sample.

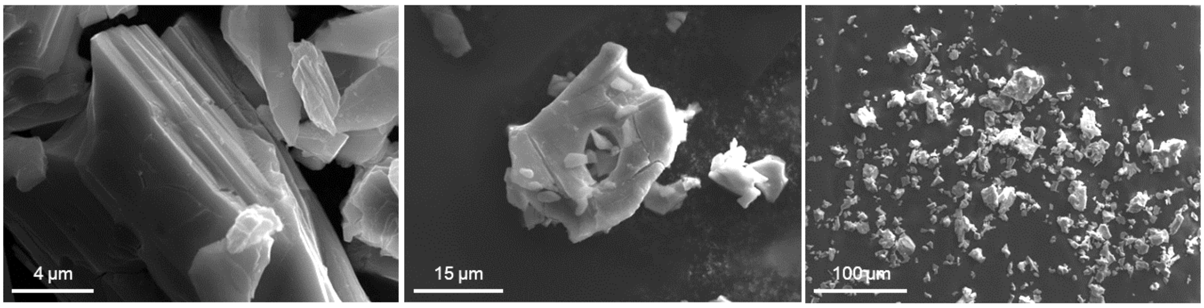

Supplementary Figure 20. SEM images of the collapsed oxysulfide phase. Secondary electron images of the Batch 2 sample at different magnification level.

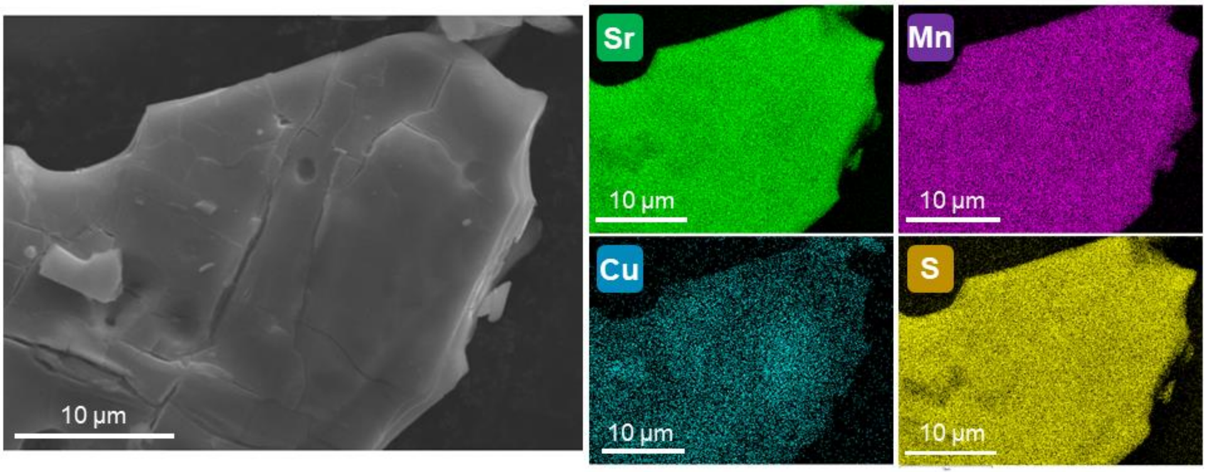

Supplementary Figure 21. Element mapping of the collapsed oxysulfide phase. (left) Secondary electron (SE) image of Batch 2 sample. See also Figure 4a-c. (right) EDX mapping acquired within the same area displaying the signal from Sr Lα_1 (green), Mn Kα_1 (purple), Cu L$\alpha_{1,2}$ (cyan) and S Kα_1 (yellow), respectively.

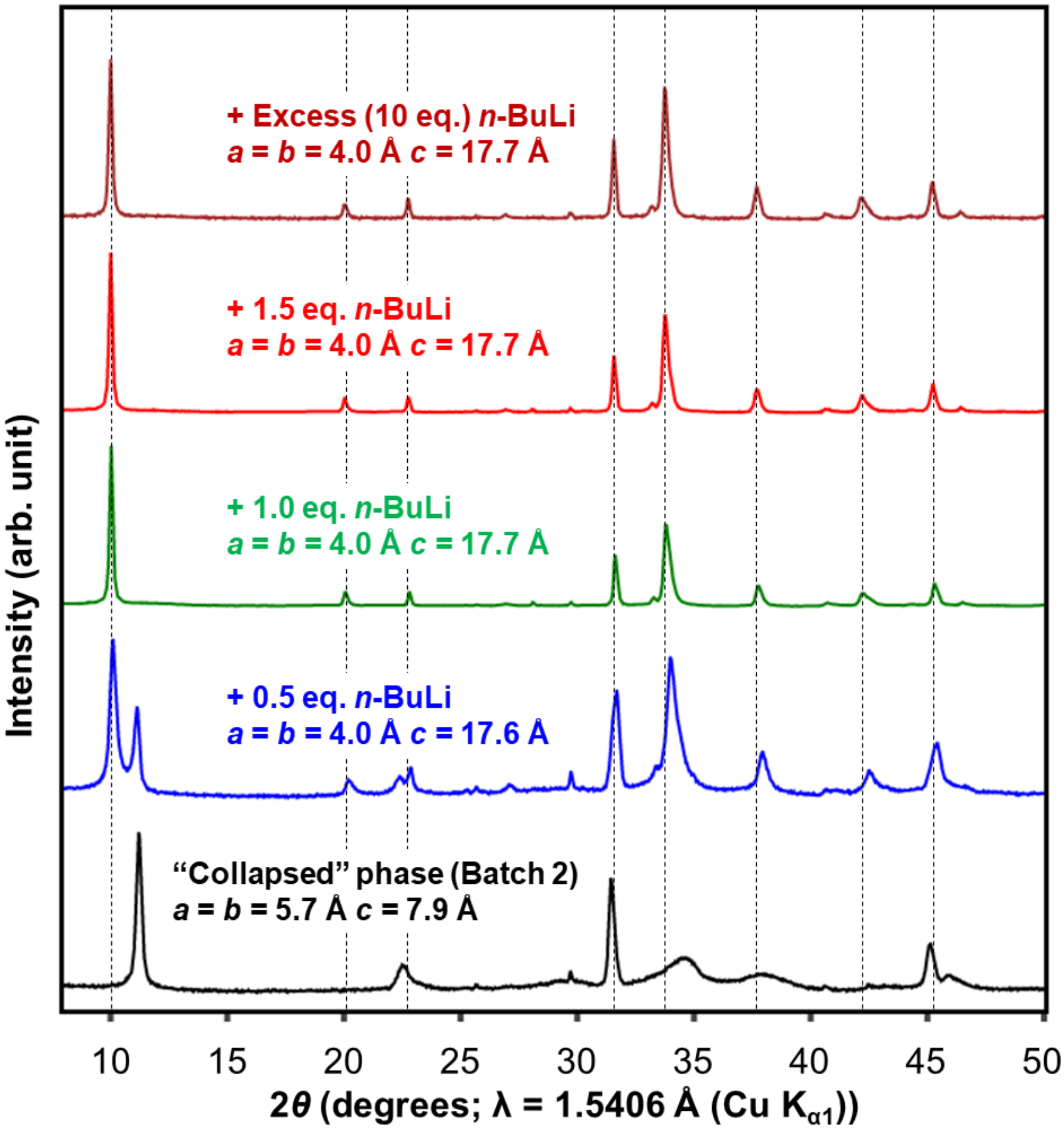

Supplementary Figure 22. X-ray diffraction (XRD) patterns after chemical re-lithiation of the collapsed oxysulfide phase. The Batch 2 sample (Composition estimated by ICP-MS: $Sr_2MnO_2Cu_{0.24}Li_{0.23}S_2$) was treated with *n*-butyllithium (*n*-BuLi) diluted to 0.1 M in anhydrous *n*-hexane at ambient temperature overnight. After addition of 1.0 eq. *n*-BuLi, the peaks from the collapsed phase were fully converted to those from $Sr_2MnO_2Li_xS_2$ phase (space group, *I*4/*mmm*) while its cell parameter (a = b = 4.00 Å, c = 17.71 Å) was significantly smaller than fully lithiated $Sr_2MnO_2Li_xS_2$ phase (a = b = 4.07 Å, c = 17.87 Å) reported by Rutt et al.[6] All experimental XRD patterns were recorded using a Bruker D8 Advance Eco X-ray diffractometer and cell parameters were estimated by Rietveld refinement.

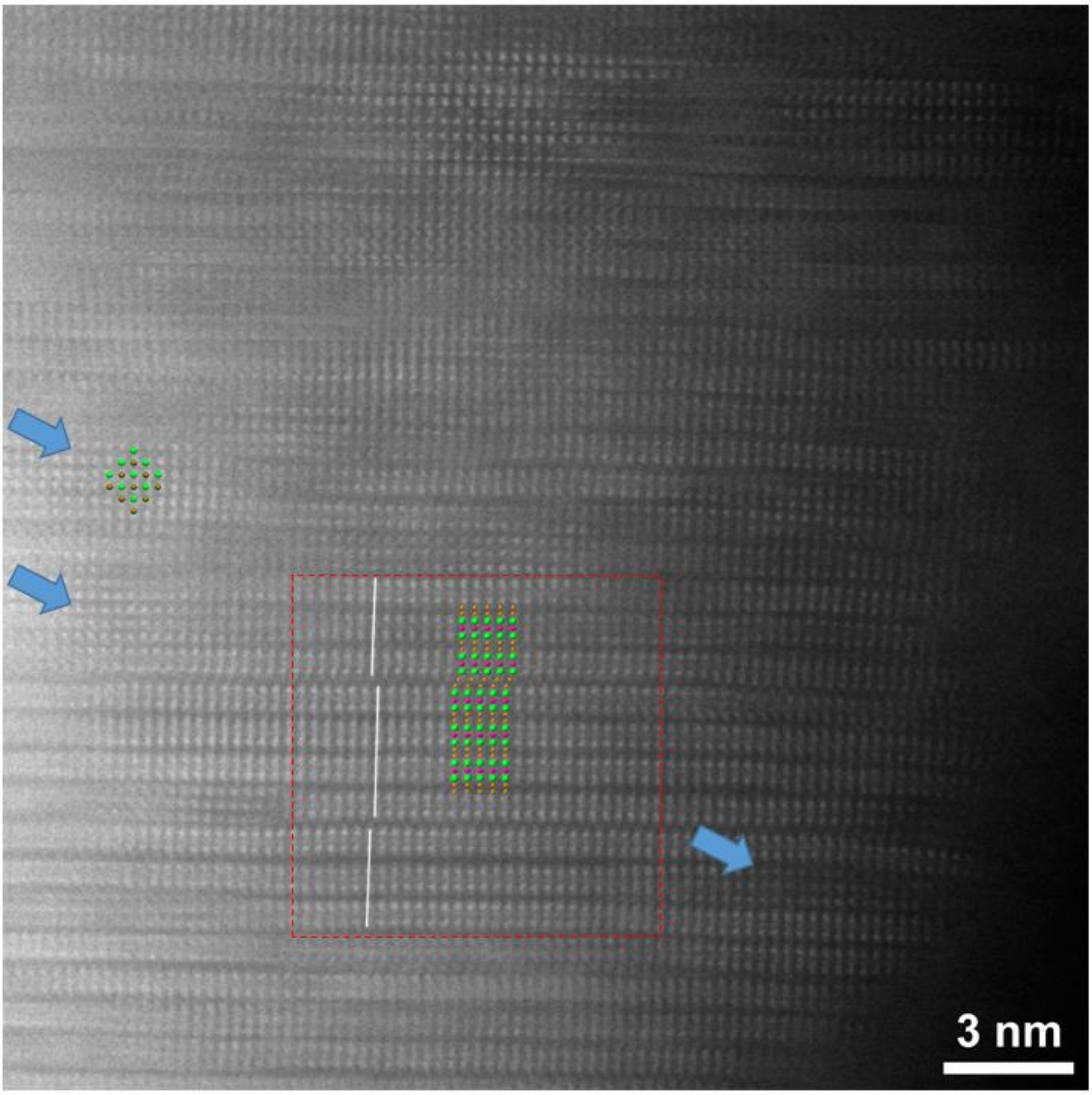

Figure 23. HAADF-STEM image of the collapsed oxysulfide phase. The image encircled by the red broken line was shown in Fig 5a. Most of the structures displayed in the image can be explained by the *C*2/*m* model of the collapsed phase seen along [100] or [010] directions. On the other hand, some other regions indicated by the blue arrows showed the basal plane of the Sr_2MnO_2 square lattice, which could be ascribed to [001] zone of the original *I*4/*mmm* structure type. The presence of that impurity phase suggests the decomposition into intercalant-rich $Sr_2MnO_2(Cu,Li)_xS_2$ phase in the same way as the thermal decomposition observed in Supplementary Figure 6.

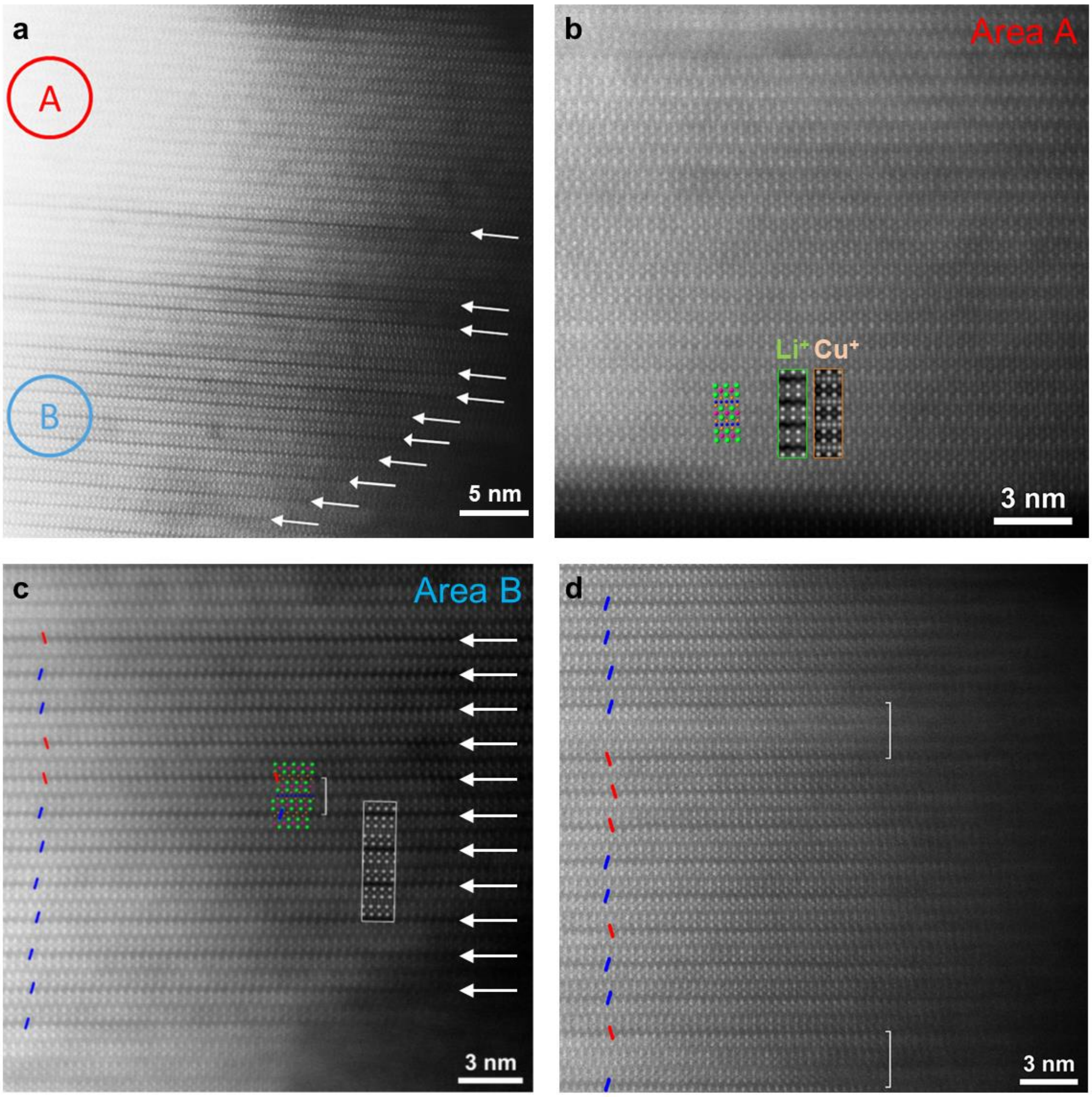

Supplementary Figure 24. HAADF-STEM images of Batch 1 sample showing the area abundant in Cu and Li-containing layers. a Overall image of the analysed area, which was comprised of two domains. Area A consisted of *I*4/*mmm* structure type similar to the parent phase $Sr_2MnO_2Cu_{1.5}S_2$. Area B features dark and thin lines indicating the metal-deficient collapsed layers (highlighted by white arrows) that intergrow with the parent-type layers. **b** Zoom-in view of Area A overlaid with [100] view of $Sr_2MnO_2Cu_{1.5}S_2$ structure,[2] whose simulated HAADF-STEM image is displayed in a brown window (right). The other simulated image in the green window (left) represents its lithiated homologue $Sr_2MnO_2Li_{1.9}S_2$.[6] **c** Zoom-in view of Area B overlaid with the intergrowth structure. In the collapsed-type layers, Sr_2MnO_2 slabs shift either left or right (highlighted by red and blue lines, respectively). In the parent-type layers, Sr_2MnO_2 slabs are stacked in body-centered manner with larger interlayer spacing. The simulated image of that intergrowth structure (white window) matched well with the observed image. **d** The other domain displayed the intergrowth structure but it also contained thicker blocks of the parent $Sr_2MnO_2Cu_{1.5}S_2$-type structure (highlighted by the brackets).

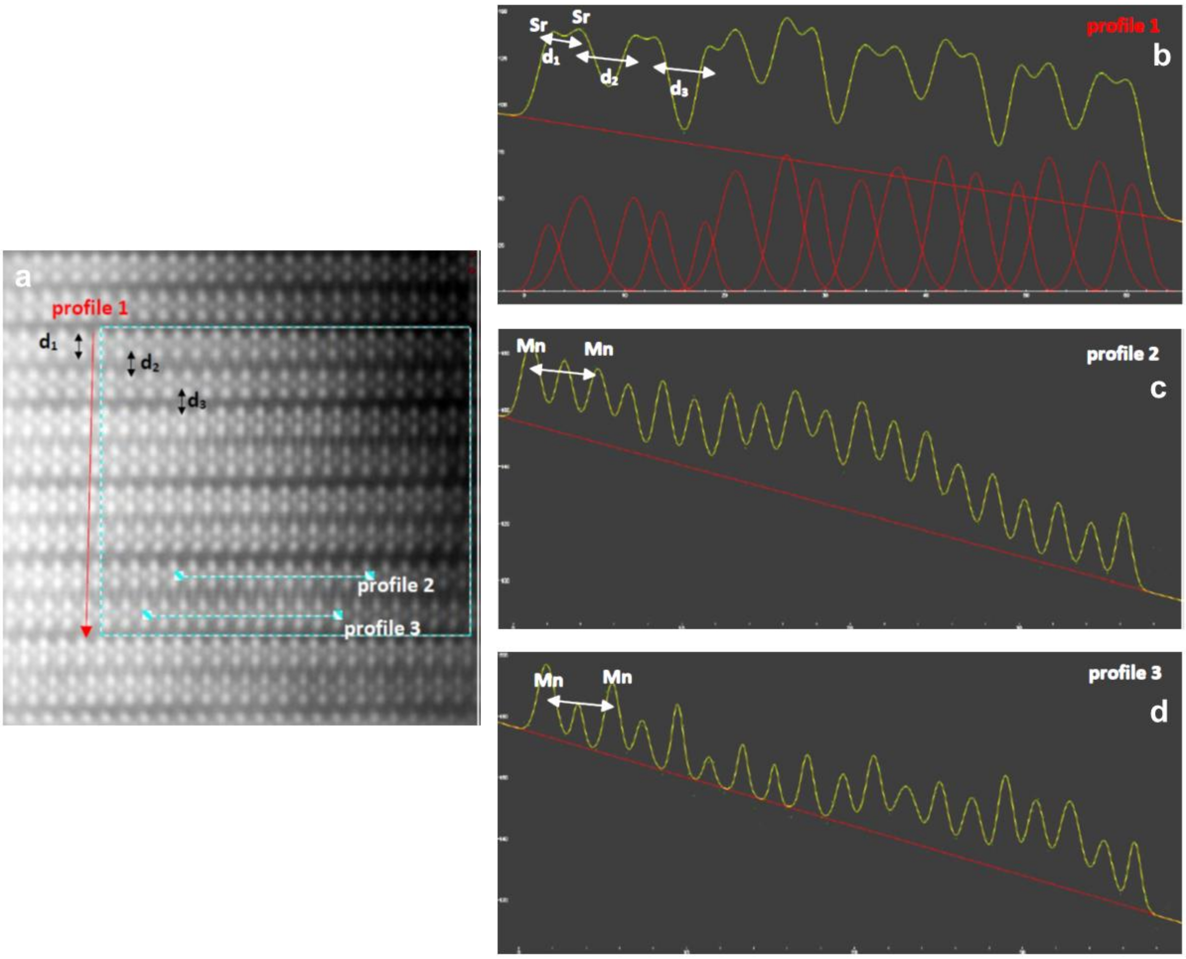

Supplementary Figure 25. Intensity profile of the HAADF-STEM image showing the intergrowth region. a Zoom-in view of the HAADF-STEM image of the Area B shown in Supplementary Figure 24c and **b** its convoluted intensity profile scanned along the line profile 1 as well as **c** along profile 2 and **d** along profile 3. Each intensity profile was deconvoluted again using Gaussian functions to extract interatomic distances. The obtained structure data was summarised in Figure 5d.

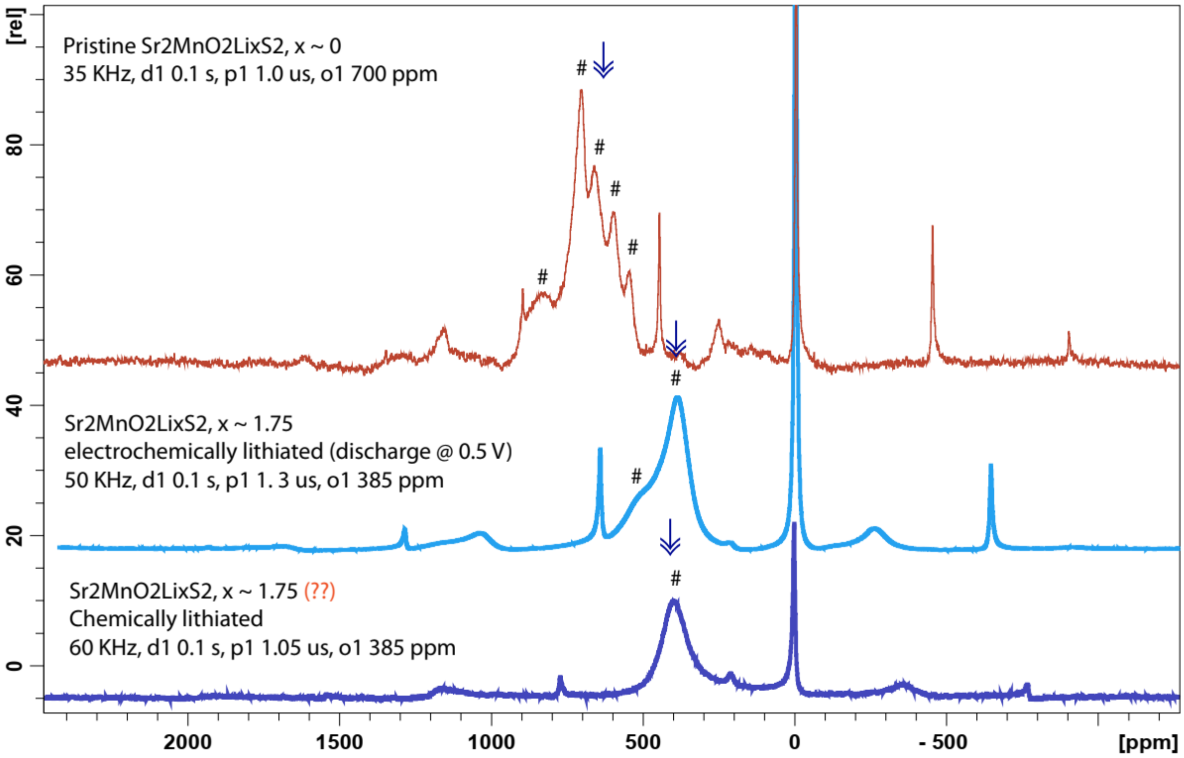

Supplementary Figure 26. ^{7}Li NMR spectra of the collapsed oxysulfide phase and its lithiated phases. The collapsed oxysulfide (brown spectrum) was lithiated either in electrochemical reduction at 0.5 V (cyan spectrum; see Figure 4d for its voltage vs Li content curve) or the reaction with 1.5 equiv. of *n*-BuLi at ambient temperature (blue spectrum; see also Supplementary Figure 22). # marks denote the main peaks from the specimen while others are from spinning sidebands.

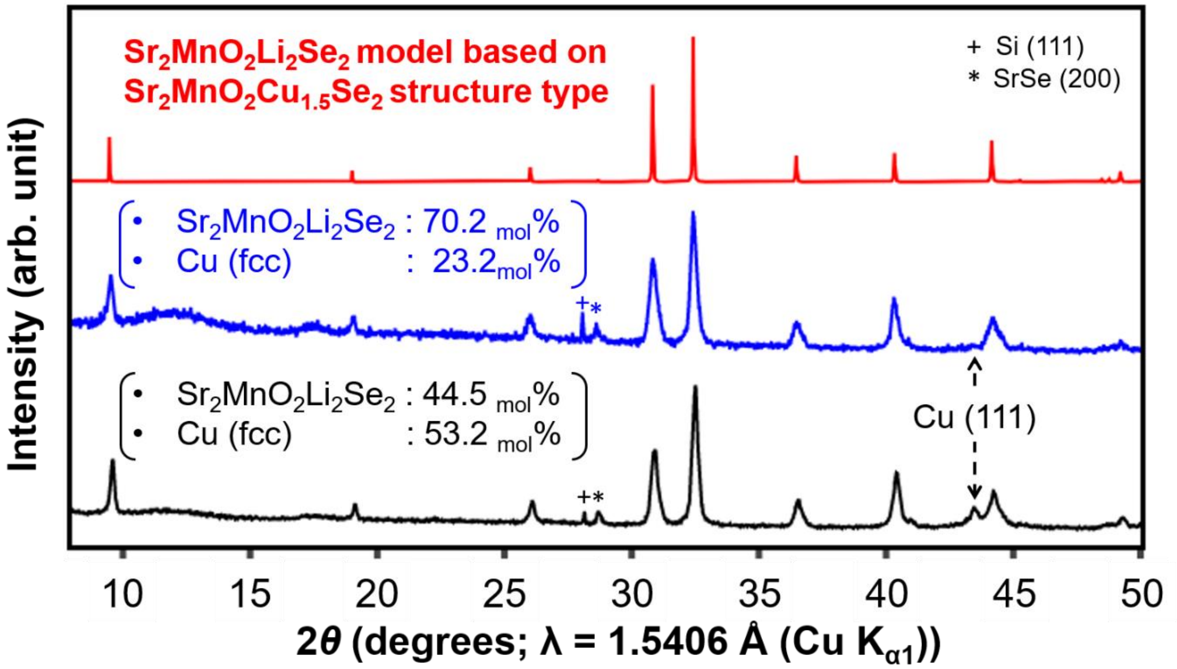

Supplementary Figure 27. X-ray diffraction (XRD) patterns after first and second lithiation for the selenide system. The black pattern was obtained after the reaction of $Sr_2MnO_2Cu_{1.5}Se_2$ with *n*-BuLi at 50 °C, showing good agreement with the simulated pattern (red) from the $Sr_2MnO_2Li_2Se_2$ model based on the $Sr_2MnO_2Cu_{1.5}Se_2$ structure type as well as the peaks from metallic Cu and minor SrSe impurity (2.3 mol%). Quantitative phase analysis by Rietveld structure refinement allowed us to estimate the molar ratio between the oxyselenide and copper to be $Sr_2MnO_2Li_2Se_2$ + 1.2 Cu. The blue pattern was obtained after dissolution of the extruded copper and subsequent treatment with *n*-BuLi. Its peaks from metallic copper were greatly reduced and Rietveld structure refinement indicated the estimated composition of $Sr_2MnO_2Li_2Se_2$ + 0.2 Cu. Both experimental XRD patterns were recorded using a Bruker D8 Advance Eco X-ray diffractometer.

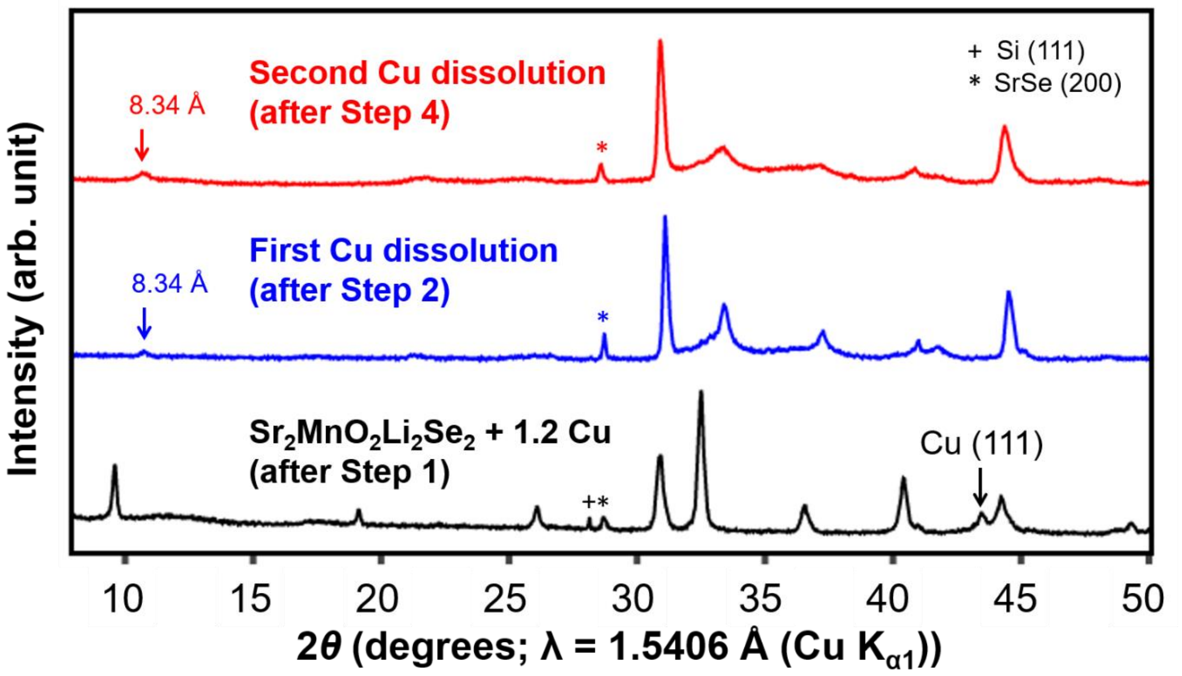

Supplementary Figure 28. X-ray diffraction (XRD) patterns after the first and second Cu dissolution. The blue XRD pattern was obtained after treating $Sr_2MnO_2Li_2Se_2$ + 1.2 Cu (the black pattern) with disulfiram, which led deintercalation of Li and Cu metals and formation of collapsed oxyselenide phase with a highly contracted cell. A similar XRD pattern was obtained when that Cu extrusion-dissolution cycle was repeated.

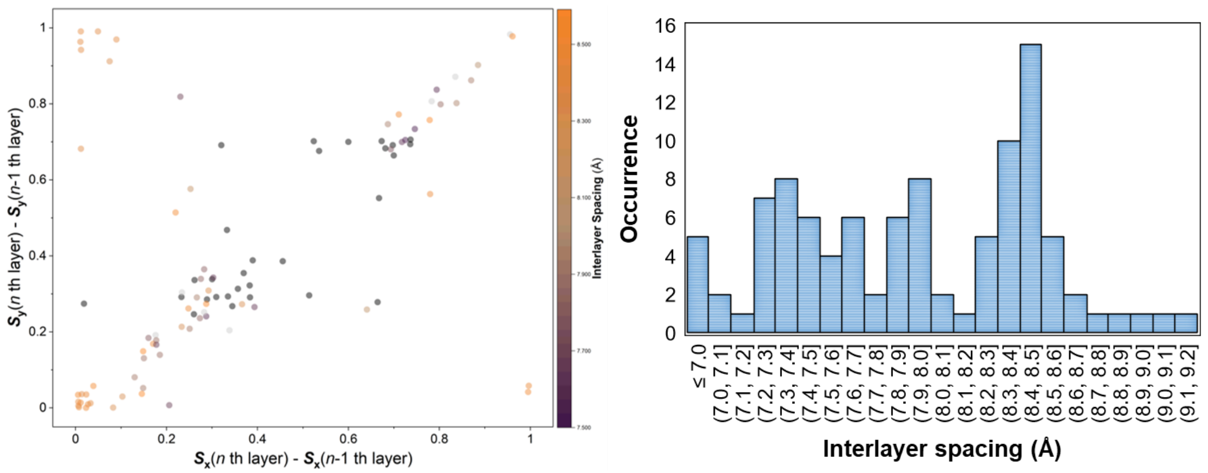

Supplementary Figure 29. Plot representing *xyz* shifts of $Sr_2MnO_2Se_2$ stacks after Rietveld refinement against Synchrotron XRD data (See Figure 7a). (Left) Scatter plots displaying sliding of each layer in *xyz* directions (S_x, S_y, S_z) that was refined as fractions of the unit cell parameters. Sliding along the stacking axis S_z was converted into interlayer spacing between adjacent $Sr_2MnO_2Se_2$ layers and shown as a function of colour. The slabs that slide toward the diagonal direction exhibit tendency towards a smaller interlayer spacing. (Right) Histogram showing the occurrence of each interlayer spacing.

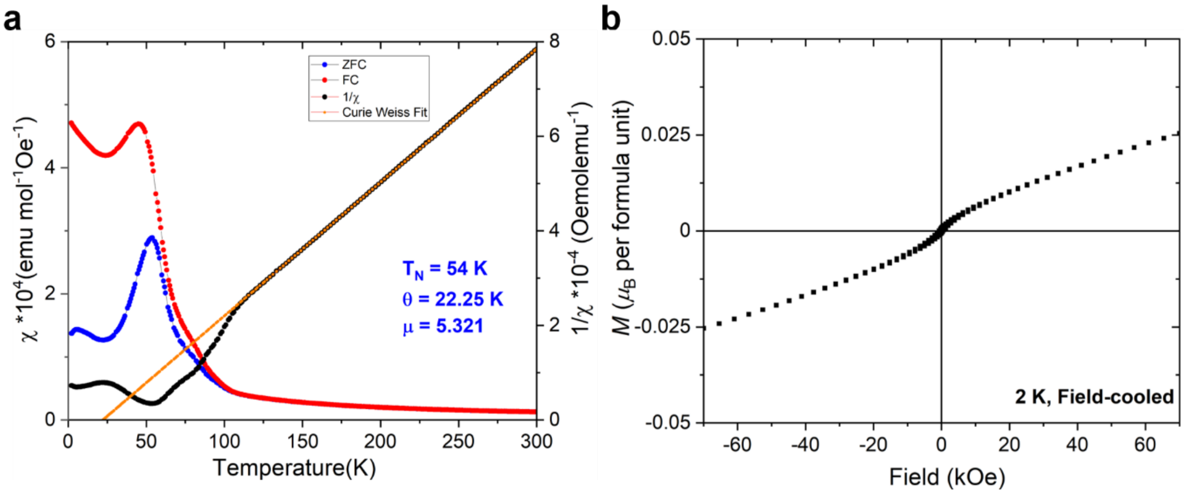

Supplementary Figure 30. χ_{mol} versus *T* plot, 1/χ_{mol} versus *T* plot and magnetisation isotherm of the collapsed oxyselenide phase. a Zero-field-cooled (ZFC, blue) and Field-cooled (FC, red) magnetic susceptibilities of the collapsed oxyselenide phase measured in an applied field of 0.01 T. Magnetisation (emu) was converted into χ_{mol} (emu mol^{-1} Oe^{-1}) supposing the tentative formula $Sr_2MnO_2Se_2$ (Molar mass = 420.1 g mol^{-1}). The linear region (150 K ≤ *T* ≤ 300 K) of the obtained 1/χ_{mol} versus *T* plot was fitted by the Curie-Weiss law $1/\chi_{mol} = (T/C) - (\theta/C)$, where *C* and *θ* represented the Curie and Weiss constants, respectively. **b** Magnetisation isotherm measured at 2 K after cooling at *H* = 100 Oe. Molar magnetisation *M* was calculated supposing the tentative formula $Sr_2MnO_2Se_2$ (Molar mass = 420.1 g mol^{-1}).

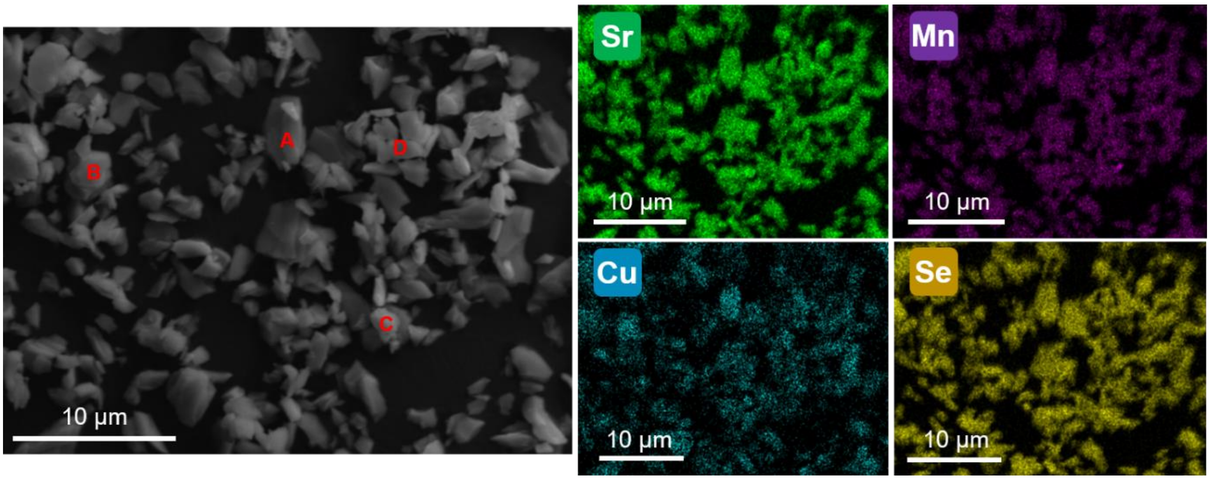

Supplementary Figure 31. Element mapping of the collapsed oxyselenide phase. (left) Secondary electron (SE) image. EDX spectra measured at different points indicated varying Cu content with Cu-rich points A and B (average Sr/Mn/Cu/Se = 2.0/1.1/0.9/2.1) and Cu-poor points C and D (average Sr/Mn/Cu/Se = 2.0/1.0/0.3/2.0) (right) EDX mapping acquired within the same area displaying the signal from Sr Lα_1 (green), Mn Kα_1 (purple), Cu L$\alpha_{1,2}$ (cyan) and Se Kα_1 (yellow), respectively.

Supplementary Tables.

Supplementary Table 1. Structural parameters of the collapsed $Sr_2MnO_2S_2$ model generated by (S_x, S_y) = (0.32, 0.32) shift[a] of the $Sr_2MnO_2S_2$ stacks.

Chemical formula			$Sr_2MnO_2S_2$	
Molar mass (g mol^{-1})			326.31	
Symmetry			Monoclinic	
Space group			*C*2/*m* (No. 12)	
a (Å)			5.6997	
b (Å)			5.6997	
c (Å)			7.9892	
β (°)			97.378	
Volume (Å^3)			257.40	
Z			2	
Density (g cm^{-3})			4.2099	
Atomic positions[b,c]				
Atom	Wyckoff	*x*	*y*	*z*
Sr	4*i*	0.0389	0	0.2159
Mn	2*b*	0	½	0
O	4*e*	¼	¼	0
S	4*i*	0.0662	½	0.3680

[a] Estimated from stacking fault analyses. See Figure 2 for details.
[b] Note that the model in Figure 2b refined relative position of $Sr_2MnO_2S_2$ slabs but structure parameters within the slabs, e.g. Mn-S and Mn-Sr distances, were fixed to the value reported for $Sr_2MnO_2Cu_{1.5}S_2$ (See Supplementary Note 1 (section 1.2) for details)
[c] When Sr-S distance of 2.995 Å obtained from the multi-parametric grid search (See Supplementary Table 2) was used, atomic coordinates of the sulphur atom was amended to (*x, y, z*) = (0.0680, ½, 0.3780), rendering its S-S distance down to 2.19 Å.

Supplementary Table 2. Approximated atomic coordinates within individual layers types observed $Sr_2MnO_2(Cu,Li)_{\sim 0.5}S_2$ crystallites defined in P1 symmetry. Layer A $a = b = 4.0303$, $c = 17.166$ Å, $\alpha = \beta = \gamma = 90°$; Layer B $a = b = 4.0303$, $c = 16.80$ Å, $\alpha = \beta = \gamma = 90°$; Layer C $a = b = 4.0303$, $c = 7.923$ Å, $\alpha = \beta = \gamma = 90°$.

	x	*y*	z (in layer)	Occ	beq	Layer
Cu1	0	0.5	0.52447	0.74	1.18	A
Cu2	0.5	0	0.52447	0.74	1.18	A
Cu3	0	0.5	0.02447	0.74	1.18	A
Cu4	0.5	0	0.02447	0.74	1.18	A
Mn1	0	0	0.27447	1	0.67	A
Mn2	0.5	0.5	0.77447	1	0.67	A
O1	0	0.5	0.27447	1	0.80	A
O2	0.5	0	0.27447	1	0.80	A
O3	0.5	0	0.77447	1	0.80	A
O4	0	0.5	0.77447	1	0.80	A
S1	0	0	0.44430	1	0.80	A
S2	0	0	0.10464	1	0.80	A
S3	0.5	0.5	0.94430	1	0.80	A
S4	0.5	0.5	0.60464	1	0.80	A
Sr1	0.5	0.5	0.37413	1	0.71	A
Sr2	0.5	0.5	0.17481	1	0.71	A
Sr3	0	0	0.87413	1	0.71	A
Sr4	0	0	0.67481	1	0.71	A
Cu1	0	0.5	0.5	0.74	1.18	B
Cu2	0.5	0	0.5	0.74	1.18	B
Mn1	0	0	0.245	1	0.67	B
Mn2	0.5	0.5	0.755	1	0.67	B
O1	0	0.5	0.245	1	0.80	B
O2	0.5	0	0.245	1	0.80	B
O3	0.5	0	0.755	1	0.80	B
O4	0	0.5	0.755	1	0.80	B
S1	0	0	0.41813	1	0.80	B
S2	0	0	0.05717	1	0.80	B
S3	0.5	0.5	0.94283	1	0.80	B
S4	0.5	0.5	0.58187	1	0.80	B
Sr1	0	0	0.65334	1	0.71	B
Sr2	0	0	0.85666	1	0.71	B
Sr3	0.5	0.5	0.14334	1	0.71	B
Sr4	0.5	0.5	0.34666	1	0.71	B
Mn1	0	0	0.5	1	0.67	C
O1	0	0.5	0.5	1	0.80	C
O2	0.5	0	0.5	1	0.80	C
S1	0	0	0.8780	1	0.80	C
S2	0	0	0.1220	1	0.80	C
Sr1	0.5	0.5	0.2841	1	0.71	C
Sr2	0.5	0.5	0.7159	1	0.71	C

Supplementary Table 3. The result of ICP-MS analyses performed for the collapsed oxysulfide phase. (See the Methods section in the main article for details)

	**Detected quantity [ng/g of sample solution][a]**			
	Sr	Mn	Cu	Li
**Blank solution**	Below DL[b]	0.2	46.2	Below DL[b]
**Batch 1**	3237.8	1087.2	454.6	16.7
**Batch 2**	3381.9	1136.5	341.3	30.8
**RSD (%)[c]**	1.03	1.41	0.84	3.12
	**Relative molar ratio before subtraction of blank solution**			
	Sr	Mn	Cu	Li
**Batch 1**	2	1.071	0.3871	0.1300
**Batch 2**	2	1.072	0.2783	0.2303
	**Relative molar ratio after subtraction of blank solution[d]**			
	Sr	Mn	Cu	Li
**Batch 1**	2	1.071	0.3478	0.1300
**Batch 2**	2	1.072	0.2406	0.2303
	**Estimated error in relative molar ratio**			
	Sr	Mn	Cu	Li
**Batch 1**	0.021	0.015	0.0035	0.0041
**Batch 2**	0.021	0.015	0.0023	0.0072

[a] Approximate concentration of dissolved powder samples: 5 μg/g of solution
[b] DL: Detection limit
[c] Relative standard deviation determined by the reference standard SPS-SW2
[d] The quantity of Mn and Cu in the blank solution were subtracted

Supplementary Table 4. The result of ICP-MS analyses performed for the collapsed oxyselenide phase. (See the Methods section in the main article for details)

	**Detected quantity [ng/g of sample solution][a]**			
	Sr	Mn	Cu	Li
**Blank solution**	Below DL[b]	10.6	49.2	Below DL[b]
**Oxyselenide**	1954.9	634.2	317.8	16.5
**RSD (%)[c]**	0.58	0.58	0.16	4.76
	**Relative molar ratio before subtraction of blank solution**			
	Sr	Mn	Cu	Li
**Oxyselenide**	2	1.035	0.4482	0.2135
	**Relative molar ratio after subtraction of blank solution[d]**			
	Sr	Mn	Cu	Li
**Oxyselenide**	2	1.017	0.3787	0.2135
	**Estimated error in relative molar ratio**			
	Sr	Mn	Cu	Li
**Oxyselenide**	0.012	0.0060	0.00070	0.010

[a] Approximate concentration of dissolved powder samples: 5 μg/g of solution
[b] DL: Detection limit
[c] Relative standard deviation determined by the reference standard SPS-SW2
[d] The quantity of Mn and Cu in the blank solution were subtracted

Supplementary Notes

Supplementary Note 1.
Rietveld refinements and Stacking Fault Analyses

Overview

General procedures of X-ray and Neutron powder diffraction measurements are outlined in the Methods section in the main article. Analyses of stacking faults were performed by using the algorithm implemented in TOPAS version 6 described by Evans and co-workers[7,8]. We adopted three different approaches to understand stacking disorders and structural diversity of the collapsed oxychalcogenides: (1) The use of a supercell in which 100 $Sr_2MnO_2S_2$ slabs are stacked and their sliding in *xyz* directions were parametrised and refined, (2) a 1000-layer supercell where each $Sr_2MnO_2S_2$ slab was allowed to take either ±0.32*a* ± 0.32*b* or zero shift relative to an adjacent layer, (3) A grid search generating various supercells that contained different amounts of the parent $Sr_2MnO_2Cu_{1.5}S_2$-type slabs, the collapsed $Sr_2MnO_2S_2$-type slabs and its intergrowth type slabs while each slab was allowed to slide in *xyz* directions relative to adjacent layers, in the similar way with the first approach. The first approach was described in Figure 2a-c and 7a-c of the manuscript while the latter two were not used for the modelling of the collapsed oxysulfide and oxyselenides shown in the main article.

The first approach allowed us to assess the most probable arrangement of two adjacent $Sr_2MnO_2S_2$ slabs and to construct the structure model of the collapsed phase as shown in Figure 2a-c. This approach could treat the disordered stacking in a statistical manner with few constraints, but the relationship between the diffraction pattern and the structural models was less evident due to the large number of independent parameters (3 × 100 degree of freedom). The second approach assumed only shifts of 0.32 along *ab* and shifts of 0 but it already gave satisfactory fitting of the XRD pattern (Supplementary Figures 13 and 14), implying that the disordered structure of the final product could be explained simply by stacking of these two different slabs. The final approach was attempted to treat the residual presence of Cu/Li intercalants in an explicit manner, by repeating numerous Rietveld

refinements with different parameters (Supplementary Figures 15 and 16). The details of each approach are described in the following.

Parametrised *xyz* sliding of a hundred $Sr_2MnO_2S_2$ slabs

The following is the detailed procedure of Rietveld refinements outlined in Figure 2a-c and Figure 7a-c. The refinement started from the large unit cell (*a* = *b* = 4.014 Å, *c* = 792.3 Å) where 100 units of $Sr_2MnO_2S_2$ slabs (Figure 2b, Space group: *P*1) are stacked with alternatingly initial offsets of 0.5*a* + 0.5*b* to mimic body centring. Structural parameters of the $Sr_2MnO_2S_2$ slab within each subcell were retrieved from the values reported for $Sr_2MnO_2Cu_{1.5}S_2$ by Gál et al.[1] normal distances from MnO_2 plane to Sr and S were fixed to 1.711 and 2.915 Å, respectively. The *c*-parameter of subcells were fixed to 7.923 Å using the peak position found on the powder XRD pattern (Figure 2a).

Each layer then has a value for S_x: offset of *x* fractional coordinate within the supercell, S_y: offset of *y* fractional coordinate within the supercell, and S_z: offset of *z* fractional coordinate within the supercell. For example, an oxygen atom in the 1st layer with an S_x=S_y=S_z = 0 will have fractional coordinates within the *a* = *b* = 4.014 Å, *c* = 792.3 Å unit cell of *x* = 0 +0, *y* = 0.5 +0, *z* = (0.5 / 100)+0, whereas the corresponding oxygen atom in the 10th layer with S_x = S_y = 0.5, S_z = 9/100 will have fractional coordinates within the unit cell of *x* = 0 +0.5, *y* = 0.5 +0.5, *z* = (0.5 / 100)+9/100. These S_x, S_y, and S_z parameters were allowed to refine within the range of $-1 \leq S_x \leq 1$, $-1 \leq S_y \leq 1$, and $S_{z(\text{initial})} -0.001 \leq S_z \leq S_{z(\text{initial})}+(8.583-7.923)/(792.3/2)$.

At the same time, the *a* and *b* parameters were refined to obtain reasonable fitting, leading to *a* = *b* = 4.0303(6) Å, which was then used for the modelling used in the other two approaches (sections 3.3 and 3.4 below). The same procedure was applied to the collapsed oxyselenide phase. The initial unit cell parameters were *a* = *b* = 4.069 Å, c = 834.3 Å but it's a and *b* parameters were then refined to 4.095 Å. *c*-parameter of subcells were fixed to 8.343 Å. Structural parameters were retrieved from the values reported for $Sr_2MnO_2Cu_{1.5}Se_2$ by Tan et al.[9]: the distances between MnO_2 planes and Sr or Se were fixed to 1.683 and 3.032 Å.

The refined S_x, and S_y, values were related to their initial values and the values for the layer below to show the offset of the layer in the *ab* plane relative to that of the preceding layer. The refined S_z values were also compared to the layer below, which can be converted to an interlayer separation. These results are plotted in Supplementary Figures 8 and 29 which were then converted into 2D Kernel plots

(shown in Figure 2c and 7b) representing density of these points by using the OriginPro software.

1000-layer supercell with predefined sliding of $Sr_2MnO_2S_2$ slabs

The result of the first approach discussed in the above section 3.2 suggests high occurrence of two structure types: *ca.* 40% of $Sr_2MnO_2S_2$ slabs show shifting by 0.32*a* +0.32*b* and 50% of them remained at the original position of body-centred stacking. Other types of relative sliding accounted for only 10% of the layers. It implies that, by imposing constraints to the type of sliding, the diffraction pattern can be explained with far fewer parameters than the first approach. In addition, it must be noted that simple Rietveld refinements were also carried out using the *C*2/*m* model (Figure 2b) without assuming any large supercell, but it gave very poor fitting to the experimental XRD data (See Supplementary Figures 11 and 12). Their refined structure seems intermediate between the parent-type *I*4/*mmm* structure and the original *C*2/*m* model (Figure 2b), as exemplified by their elongated S-S bond distances $d_{S\text{-}S}$ around 2.6 Å. This second approach with predefined sliding is also to give a qualitative explanation about why such faultless models did not give good fitting.

Therefore, we modelled a large, low-parameter supercell similar to that shown in Figure 2b, but with each $Sr_2MnO_2S_2$ slab only allowed to take either a shift of 0 (maintaining body centring) or a shift by a predefined value $s = S_x = S_y$ along *ab*. When a shift of *s* along *ab* occurs, it can occur in one of four possible directions, as shown in Supplementary Figure 13. This disorder, akin to twinning, is observed in the STEM images of Figure 5 as marked by the red and blue lines. Note that the lack of depth perception in the STEM image means each blue or red line could itself be one of two possible directions of S-S bond formation, with the layer above moved either towards or away from the viewer relative to the layer below.

In this approach, 1000-layer supercells can be built from $Sr_2MnO_2S_2$ slabs using the following 6 predefined parameters: *s*: the layer shift along *ab*, P_0: the probability of the next layer being unshifted relative to the one below, P_1: the probability of the next layer being shifted by +*s* along a and +*s* along *b*, P_2: +*s* along *a* and -*s* along *b*, P_3: -*s* along *a* and +*s* along *b*, and P_4: -*s* along *a* and -*s* along *b*. If P_1 is equal to 1.0 and all other probabilities are 0, all the diffraction peaks are sharp. Introducing non-zero P_0 and/or $P_{2\text{-}4}$ will largely maintain the sharpness of the *hk*0 and 00*l* reflections, but give broadening of the remaining peaks, like that observed in the diffraction data. Using

only these parameters it is possible to demonstrate the difference between the faultless model displaying poor fitting (See Supplementary Figures 11 and 12) and the stacking fault model, as well as showing that the small-supercell high-parameter stacking fault model (described in section 3.2 above) can be largely replicated with a large supercell governed by a small number of parameters.

The faultless model seen in Supplementary Figures 11 and 12 can be closely replicated by introducing a single shift of s = +0.17 along a and b for every layer. This gives an effective beta angle of 103.6°, matching the refined structure in Supplementary Figure 12 closely apart from the constraint that $a = b$. Introducing only twin-type faults by increasing $P_{2\text{-}4}$, with $P_1 = (1-P_0)*[1-(P_2+P_3+P_4)]$ and $P_0 = 0$ gives some of the required broadening to the peakshapes without the need for anisotropic peak broadening parameters. This gives the fit in Supplementary Figure 14a, which, like the fit in Supplementary Figure 12, gets the peak positions in the 17-22° region correct but fails to match many other features including the broad peak at 30.5°. If instead, s = 0.32 but P_0 is 0 then it is not possible to get the peak positions in the 17-22° region correctly centred, as shown in Fig S13b: the predicted maxima lie either side of the observed peaks. If s = 0.17 but P_0 is increased, the maxima in the 17-22° region shift in towards the centre and no overall improvement can be made to the fit in this region (Supplementary Figure 13c), but if s = 0.32 is used then introduction of P_0 to around 0.40 will give a reasonable fit to the data, matching the features in 17-22° region, the sharp 00l and hk0 peaks, and the broad peak at 30.5° (Supplementary Figure 13d).

The fit in Supplementary Figure 13d was arrived at by choosing s = 0.32 and P_0 = 0.4 based on the small supercell refinement, then trial and error adjustment of $P_{0\text{-}4}$ to give a similar degree of peak broadening to the observed. Note that it does not include any variation in the layer heights or correlation between those layer heights and the P_0 parameter, but demonstrates the initial ‘small, high parameter’ supercell is accurately extracting the parameter s, and not just fitting the data well because of its large number of parameters.

Although the fitting in Supplementary Figure 14d already explain the diffraction in a qualitative manner, its quality was not as good as the one from the first approach shown in Figure 2b. The fit could be further improved by performing an iterative grid search over the 6 parameters, such as that performed by Bette et al.[10] It would also be possible to devise parameters defining the probability of a larger layer separation,

the size of the separation, and the correlation between that and separation P_0 versus P_{1-4}. We can imagine a second scenario in which instead of P_{1-4} being the same regardless of the preceding layer, the preceding layer gives a higher probability that the next layer has the same shift. Nevertheless, we prioritised the effect of Cu/Li intercalated layers over these secondary effects when such a grid search was performed (see the next section), as the former is likely to give more impact on the diffraction pattern.

Multi-parametric grid search generating various supercells

The third approach also considers the presence of the oxysulfide slabs still containing Cu and/or Li intercalants. In this approach, a supercell is described by stacking of three distinct types of slab: A-type representing the parent structure, C-type representing the collapsed phase, and B-type representing the intergrowth between them (Supplementary Figure 15, see Supplementary Table 2 for their atomic coordinates). The A-type structure was taken from the reported $Sr_2MnO_2Cu_{1.5}S_2$ model[2] and C-type structure was equivalent to the $Sr_2MnO_2S_2$ slab used in the first approach, whose atomic parameters were however subject to refinement later (*vide infra*). The B-type structure's unit cell was given a cell parameter *c* of 16.8 Å based on the indexing from that region's electron diffraction pattern. The cell parameter *a* of the three structures was required to be the same in order to stack the three cells together. This is likely to be a valid assumption since the *a* cell parameter is equal to twice the Mn-O bond length, which will be largely unchanged between the structure types since the XANES shows little effect on the Mn oxidation state. The *a* cell parameter was initially chosen to be that of the parent phase (4.0139 Å) and later refined to 4.0303(6) Å. The coordinates were then chosen such that the bond lengths in the Cu and vacant layers were similar to those in the A-type and C-type structures.

Then, we attempted to model the data using parameters to define the probability of transitioning between A-type, B-type and C-type stacking, the B-type and C-type layers themselves contain twin type faults. A custom code was written to generate supercells which could be run in a batch mode Rietveld refinement. Each supercell was generated with A, B and C type layers based on the following set of parameters: *s:* the *ab* shifting for both B-type and C-type layers, P_{AA},P_{AB}…-P_{CC}: 9 parameters defining the probability of one type of layer following the preceding type of layer, P_{1-4}: 4 parameters to give the probabilities of ++, +-, -+, or -- shifts of *s* along *ab* from any

given B-type or C-type layer, and $P_{1'\text{-}4'}$: 4 parameters to give the probabilities of ++, +-, -+, or -- shifts of *s* along ab based on the preceding layer (run in a separate iteration to $P_{1\text{-}4}$).

It was necessary to reduce the number of parameters so $P_{1\text{-}4}$ and $P_{1'\text{-}4}$' were each reduced to one parameter P_1, with $P_{2\text{-}4}$=(1-P_1)/3. The 9 P_{AA}-P_{CC} parameters were reduced to two parameters based on the observed composition in the EDX. The total composition contains ~ 1/3 of the Cu/Li occupancy of the parent structure, suggesting 1 in 3 of the sulfide layers are occupied by Cu/Li. If only A and C type layers were present this would mean a 1:2 ratio, or if only B and C type, a 2:1 ratio. If A-, B-, and C-types are all present then for any given fraction of A- type layers a parameter, P_{ABC}, can be used to denote the remaining fractions of B-type and C-type layers that are necessary to match the EDX composition.

A second parameter P_m is also required to relate the ratio parameter P_{ABC} to the probability of transitioning from each layer type, where P_m denotes the amount of intergrowth between the layer types, e.g. if the ratio of A to C is 1:2 this could be given by P_{AA} = 0.98, P_{AC} = 0.01, P_{CC} = 0.99, P_{CA} = 0.01, (a low P_m) or by P_{AA} = 0.5, P_{AC} = 0.5, P_{CC} = 0.75, P_{CA} = 0.25, (a high P_m). The combination of P_{ABC} and P_m can be used to generate the probabilities of transitions between phases P_{AA}-P_{CC}. Since this is a fairly hard constraint, some randomness was introduced by also taking the $P_{AA\text{-}CC}$ probabilities and adding or subtracting a random number to them before renormalising. This randomisation was performed N_r times.

In all this leaves just four parameters to be searched over: *s*, P_{ABC}, P_m, and P_1 or separately *s*, P_{ABC}, P_m, and $P_{1'}$. Iterative grid searching for a desirable combination of these parameters was performed e.g. over: 3 values of *s* = 0.28, 0.32, and 0.36, 6 values of P_{ABC} from all A- and C-type to all B- and C-type, 6 values of P_m from low mixing to high mixing, and 6 values of P_1 or $P_{1'}$=0.99, 0.85, 0.7, 0.55, 0.4, and 0.25, with randomisation being performed on the transition probability matrix N_r=5 times. This generates 3×6^3×2×5=6480 supercells, which can each be refined against the data.

There are also more traditional parameters within the Rietveld refinement that can be chosen to be refined (or not). The arrangement of the layers cannot be adjusted for each supercell during refinement, but more other components of the refinement can, such as the background (initially kept fixed as a user drawn line, but some freedom can be introduced with a polynomial), the peakshape (initially kept fixed as a

single Lorentzian crystallite size broadening contribution but this can be refined within limits), the atomic coordinates within the layer (initially fixed but can be refined with limits to keep coordination environments symmetric where appropriate and bond distances reasonable), copper occupancy within the Cu/Li containing layers (initially set to the parent phase value but can be allowed to refine within limits), and thermal displacement parameters (initially kept fixed but can be refined within appropriate limits). The 6480 distinct supercells were used in refinements four times with increasing freedom in the additional Rietveld parameters, but none could provide an adequate fit to the data. The failure of this modelling was identified as an incompatibility between the P_m parameter and the sharpness of the first 00*l* peak versus the broadness of the other features in the pattern.

If there is an intergrowth between A-type and C-type layers, but very little mixing – i.e. effectively 1000 layers of A followed by 1000 layers of C, then the pattern is effectively that of a two-phase sample, with individual contributions from each region, as shown by in Supplementary Figure 16. Supplementary Figure 16c is essentially a weighted combination of Supplementary Figures 16a and 16b. As the P_m mixing value is increased, the two 00*l* (8.58 Å and 7.92 Å) contributions will each broaden, then start to merge to become a broader, single average peak with a position at the weighted average of the two, shown in Supplementary Figure 16d. The observed sharpness of the 1st 00*l* peak indicates the mixing is sparse, and there are large regions of regular layers separated by 7.923 Å, with little intergrowth of larger Cu containing layers. This however is inconsistent with the fact that intergrowth of unshifted, body centred layers is necessary to match the broad features latter in the pattern, as was shown in Supplementary Figure 14d. If a highly intergrown structure is assumed then a better fit to some of the broader features can be achieved, but the 1st peak position becomes displaced. It is possible to shift the position of the merged 00*l* by assuming a lower *c* cell parameter than 7.92 Å for the C-type layers (e.g. 7.7 Å), however the 00*l* peak remains very broad relative to the observed and the fit elsewhere in the pattern isn’t entirely satisfactory as shown in Supplementary Figure 16e. The same scenario is encountered when introducing the B-type phase as well. Better fits can be achieved by combining the two scenarios: one stacked phase with little intergrowth and one stacked phase that’s highly intergrown as shown in Supplementary Figure 16f. Despite the multitude of factors taken into account in this approach, the best fit shown Supplementary Figure 16f was not as good as the one produced by the first approach

(Figure 2b). Further improvement might be achieved by introducing more phases, which is suggestive of inhomogeneity across the crystallites with a range of compositions being present: possibly driven by differing levels of penetration of the deintercalation reagent (disulfram) and solvent with respect to the surfaces and depth of the crystallites.

Supplementary References

1. Blandy, J. N., Abakumov, A. M., Christensen, K. E. et al. Soft chemical control of the crystal and magnetic structure of a layered mixed valent manganite oxide sulfide *APL Mater.* **3**, 041520 (2015).

2. Gal, Z. A., Rutt, O. J., Smura, C. F., Overton, T. P., Barrier, N., Clarke, S. J. & Hadermann, J. Structural Chemistry and Metamagnetism of an Homologous Series of Layered Manganese Oxysulfides. *J. Am. Chem. Soc.* **128**, 8530-8540 (2006).

3. Smith, R. I., Hull, S., Tucker, M. G., Playford, H. Y., McPhail, D. J., Waller S. P., & Norberg, S. T. The Upgraded Polaris Powder Diffractometer at the ISIS Neutron Source. *Rev. Sci. Instrum.* **90**, 115101 (2019).

4. P. W. Stephens, Phenomenological model of anisotropic peak broadening in powder diffraction. *J. Appl. Cryst.* **32**, 281-289 (1999).

5. Sasaki, S., Lesault, M., Grange, E., Janod, E. Corraze, B., Cadars, S., Caldes, M. T., Deudon, C. G., Jobic, S. & Cario, L. Unexplored reactivity of $(S_n)^{2-}$ oligomers with transition metals in low-temperature solid-state reactions. *Chem. Commun.* **55**, 6189-6192 (2019).

6. Rutt, O. J., Williams, G. R. & Clarke, S. J. Reversible lithium insertion and copper extrusion in layered oxysulfides. *Chem. Commun.* 2869-2871 (2006).

7. Ainsworth, C. M., Lewis, J. W., Wang, C.-H., Coelho, A. A., Johnston, H. E., Brand, H. E. A. & Evans, J. S. O. 3D Transition Metal Ordering and Rietveld Stacking Fault Quantification in the New Oxychalcogenides $La_2O_2Cu_{2-4x}Cd_{2x}Se_2$. *Chem. Mater.* **28**, 3184-3195 (2016).

8. Coelho, A. A., Evans, J. S. O. & Lewis, J. W. Averaging the intensity of many-layered structures for accurate stacking-fault analysis using Rietveld refinement. *J. Appl. Cryst.* **49**, 1740-1749 (2016).

9. Tan, S. G., Lei, H. C., Lu, W. J., Tong, P., Li, L. J., Lin, S., Huang, Y. N., Huang, Z. H., Liu, Y., Zhao, B. C. & Sun, Y. P. Layered oxyselenides $Sr_2Co_{1-x}Mn_xO_2Cu_{2-\delta}Se_2$: The evolution of magnetic properties tuned by the competed interactions. *J. Alloy Compd.* **598**, 171-176 (2014).

10. Bette S., Hinrichsen, B., Pfister, D., Dinnebier, R. E. A routine for the determination of the microstructure of stacking-faulted nickel cobalt aluminium hydroxide precursors for lithium nickel cobalt aluminium oxide battery materials. *J. Appl. Cryst.* **53**, 76-87 (2020).
